# A common terminology for the external anatomy of centipedes (Chilopoda)

**DOI:** 10.3897/zookeys.69.737

**Published:** 2010-11-18

**Authors:** Lucio Bonato, Gregory D. Edgecombe, John G.E. Lewis, Alessandro Minelli, Luis A. Pereira, Rowland M. Shelley, Marzio Zapparoli

**Affiliations:** 1 Università di Padova, Dipartimento di Biologia, via Ugo Bassi 58 B, I-35131 Padova, Italy; 2 Natural History Museum, Cromwell Road, London SW7 5BD, United Kingdom; 3 Somerset County Museum, Taunton Castle, Castle Green, Taunton, Somerset TA1 4AA, United Kingdom; 4 Universidad Nacional de La Plata, Facultad de Ciencias Naturales y Museo, Paseo del Bosque s.n., AR-1900 La Plata, Argentina; 5 Research Laboratory, North Carolina State Museum of Natural Sciences, 4301 Reedy Creek Road, Raleigh, North Carolina 27607, U.S.A.; 6 Università degli Studi della Tuscia, Dipartimento di Protezione delle Piante, via S. Camillo de Lellis, I-01100 Viterbo, Italy

**Keywords:** Chilopoda, morphology, terminology

## Abstract

A common terminology for the external morphological characters of centipedes (Chilopoda) is proposed. Terms are selected from the alternatives used in the English literature, preferring those most frequently used or those that have been introduced explicitly. A total of 330 terms are defined and illustrated, and another ca. 500 alternatives are listed.

## Introduction

This contribution is intended to propose a common terminology for the external morphological characters of centipedes (Chilopoda).

Students still use different terms to describe the same or similar structures in centipedes, even limiting our survey to papers in English. Consequently, the terminology is heterogeneous, redundant, and sometimes ambiguous. The lack of standardization hinders comparative analysis and integration of information scattered through the literature, and discourages new students from undertaking taxonomic and morphological investigations on this arthropod group.

Efforts to revise the terminology have been rare and limited to either particular character sets or selected chilopod sub-groups, as exemplified by the terms for integumental projections discussed by Crabill (1960a) and those proposed for major taxonomic characters in Scolopendromorpha (Lewis et al. 2005). A common English terminology encompassing all external features and applicable to the Chilopoda as a whole has never been proposed.

## Methods

The terminology recommended here encompasses major structural features of the body and all external characters recognized under light microscopy. Internal characters are not addressed herein, because the terminology in use is more consistent and uniform. We also exclude fine structural details, including those of peristomatic structures (epipharynx and hypopharynx), because they have been documented only recently, by histology and scanning electron microscopy, so that a consistent terminology is available (Edgecombe and Giribet 2006; Koch and Edgecombe 2006, 2008). Our recommended terminology mainly focuses on adult morphology of extant chilopods, but is intended to be applicable to other post-embryonic stadia and to extinct taxa as well.

We considered all publications in English dealing with centipedes since Lewis’ (1981) treatise on chilopod biology (the most recent, comprehensive synthesis on the morphology of this group in English) and a selection of older works also in English (listed in the additional file: Pre-1981 publications) that seemed most relevant for the morphological terminology. We omitted XIX century publications, because their terminologies were often based on erroneous or unwarranted homologies with other arthropods and have long been superseded. We retrieved all applicable terms and assessed counterparts.

To maximize future applicability, alternative criteria of selection have been discussed with authors who are either currently the most active centipede systematists publishing descriptions in English and/or have already addressed issues of terminology standardization. In order to identify and recommend a single term for each character, we applied the following criteria: (i) we selected a term already used in the literature, except when all alternatives are either ambiguous or inconsistent with other selected terms; (ii) among alternatives, we selected either the term used most frequently (by most authors and/or in most publications) or the one explicitly introduced and defined by an influential author; (iii) we applied minor emendations to selected terms (in endings, prefixes, hyphenations between elements of compound words) when necessary for consistency and uniformity. We refrained from revising the terminology based on homology hypotheses with other arthropods (Edgecombe 2008), because many relationships remain under debate.

Major anatomical differences exist between the six centipede orders, five extant - Scutigeromorpha, Lithobiomorpha, Craterostigmomorpha, Scolopendromorpha, and Geophilomorpha - and one extinct, Devonobiomorpha. Morphological and taxonomical investigations by different authors have sometimes been and still are limited to single orders, leading to different terminological traditions. While we propose a consistent terminology for the entire class, we specify the order(s) to which each term is applicable to facilitate usage by students interested in single orders; when no orders are specified, it is meant that the term is applicable to all orders; when an order is specified, it is meant that the term is applicable to at least some taxa in the order.

## Results

After reviewing the relevant literature as explained above, we retrieved roughly 830 terms that apply to 330 anatomical features. By applying the criteria described above, we obtained the recommended terminology presented herein.

Terms for surface depressions and projections are provided in Tables 1–2. Those indicating the arrangement of these and other features are given in Table 3. Terms recommended but not defined because of general use in Arthropoda include: appendage, arthrodial membrane, article, articulation, condyle, cuticle, head, pleurite, sclerite, segment, sternite, telopodite, tergite, and trunk. All other recommended terms are listed below. They are arranged first in anterior to posterior anatomical sequence and then in hierarchical-structural order (whole to part). The singular is in bold, and specifications that may be omitted are in parentheses. For each recommended term, we give the following: whenever suitable, the plural preceded by a slash; in the case of taxonomically restricted usage, the order(s) to which it applies (in brackets); a synthetic definition; reference to an illustration; whenever suitable, reference(s) to publication(s) where the term was defined; synonymous terms (introduced by “Syn.”; the plural form preceded by a slash; listed alphabetically, without an implicit ranking). An alphabetical index of all recommended and synonymous terms is provided in the additional file: Analytical index. Abbreviations for orders are: Cra (Craterostigmomorpha), Dev (Devonobiomorpha), Geo (Geophilomorpha), Lit (Lithobiomorpha), Sco (Scolopendromorpha), and Scu (Scutigeromorpha).

**Table 1.** Terms recommended for different kinds of impressions on the body surface.

**recommended term**/plural	features	source	alternative terms employed
**suture**/sutures	linear; sometimes corresponding to the seam between two immovable sclerites	Lewis et al. 2005: 3	hinge line/lines, sulcus/sulci
**sulcus**/sulci	shallow, elongated; in a sclerite	Lewis et al. 2005: 3	furrow/furrows, stria/striae
**depression**/depressions	shallow, large, not elongated; in a sclerite	Lewis et al. 2005: 2, 5	gutter/gutters
**fossa**/fossae	deep, large, elongated; in a sclerite or between two sclerites	Eason 1964: 278. Barber 2009: 204	pit/pits
**punctum**/puncta	point-like; in a sclerite	-	-
**setal socket**/sockets	deep, rounded; corresponding to a seta	-	setal alveolus/alveoli

**Table 2.** Terms recommended for different kinds of projections on the body surface.

**recommended term**/plural	features	source	alternative terms employed
**tubercle**/tubercles	non-articulated, stout, usually rounded	-	-
**spinous process**/processes	non-articulated, large, pointed	Lewis et al. 2005: 5	-
**spine**/spines	non-articulated, small, pointed	Crabill 1952: 204. Crabill 1962: 399. Würmli 1974: 93. Edgecombe and Giribet 2006: 509	spina/spinae
**spinula**/spinulae	non-articulated, very small, pointed	Würmli 1974: 93. Edgecombe and Giribet 2006: 509	spinule/spinules
**hair**/hairs	non-articulated, slender	Würmli 1974: 93	-
**spicula**/spiculae	non-articulated, spike-like	Würmli 1974: 93	hairlike spine/spines, spiculum/spicula
**seta**/setae	articulated at tde base, slender	Crabill 1952: 204. Crabill 1960a: 14	bristle/bristles, hair/hairs, trichoid sensillum/sensilla
**spur**/spurs	articulated at tde base, spine-like	Crabill 1952: 204. Crabill 1960a: 14. Crabill 1962: 399. Lewis et al. 2005: 5	-
**spine-bristle**/spine-bristles	articulated at tde base, slender, large, covered witd short spines proximally that elongate into a fluted ornament distally	Würmli 1974: 93	acicular seta/setae, macroseta/macrosetae, spinoseta/spinosetae
**sensillum**/sensilla	articulated at tde base, shape various, sensorial function	-	sensory seta/setae

**Table 3.** Terms recommended for indicating the pattern of different elements.

recommended term	elements	alternative terms employed
**areolation**	scutes, on an area of the surface	reticulation
**setation**	setae, on an area of the surface	chaetotaxy, vestiture
(coxosternal) **dentition**	teeth, on the anterior margin of the forcipular coxosternite (Lithobiomorpha)	-
**plectrotaxy**	spurs, on the legs (Lithobiomorpha)	armature, spinulation, spurulation

### cephalic capsule

**Figures 1–8.**
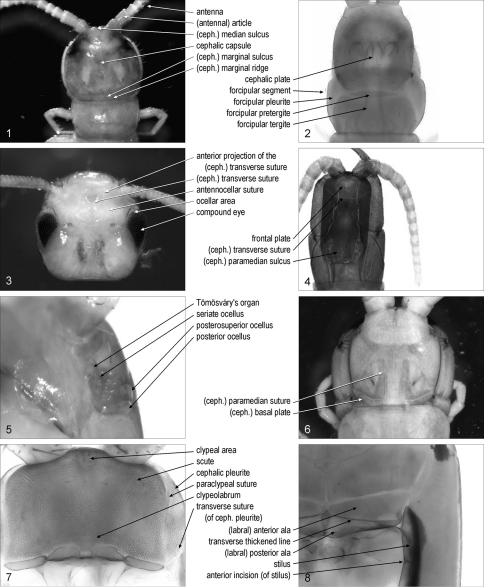
**1** anterior part of body, dorsal, Lamyctes emarginatus **2** anterior part of body, dorsal, Geophilus carpophagus **3** head, dorsal, Scutigera coleoptrata **4** anterior part of body, dorsal, Mecistocephalus guildingii **5** left part of cephalic capsule, ventro-lateral, Lithobius dentatus **6** anterior part of body, dorsal, Cormocephalus gervaisianus **7** anterior part of cephalic capsule, without maxillary complex and mandibles, ventral, Ribautia centralis **8** left part of cephalic capsule, without maxillary complex and mandibles, ventral, Mecistocephalus togensis. Abbreviations: ceph., cephalic.

**cephalic capsule**: integument of the head to the exclusion of its appendages. Fig. 1. Syn.: head capsule

**cephalic plate**: [Cra, Dev, Geo, Lit, Sco] dorsal side of the cephalic capsule. Fig. 2. Syn.: cephalic shield, head plate, head shield

(cephalic) **median sulcus**: [Lit, Scu] mid-longitudinal sulcus on the anterior part of the cephalic capsule. Fig. 1. Syn.: (cephalic) median furrow

(cephalic) **transverse suture**: transverse suture on the anterior part of the dorsal side of the cephalic capsule. Figs 3–4. Syn.: cephalic suture, frontal line, frontal sulcus, frontal suture

**anterior projection**/projections **of the** (cephalic)**transverse suture**: [Scu] one of the paramedian sutures projecting anteriorly from the cephalic transverse suture. Fig. 3

**antennocellar suture**/sutures: [Cra, Lit, Scu] one of the paired sutures on the antero-lateral parts of the cephalic capsule. Fig. 3. Crabill 1961a: 131

**antennal branch**/branches **of antennocellar suture**: [Cra, Lit, Scu] part of the antennocellar suture, anterior to the cephalic transverse suture. Syn.: anterior portion/portions of antennocellar suture

**ocellar branch**/branches **of antennocellar suture**: [Cra, Lit, Scu] part of the antennocellar suture, posterior to the cephalic transverse suture. Syn.: posterior portion/portions of antennocellar suture, posterior limb/limbs of (cephalic) transverse suture

**frontal plate**: anterior part of the dorsal side of the cephalic capsule, delimited posteriorly by the cephalic transverse suture. Fig. 4. Syn.: frons

**ocellar area**/areas: [Lit, Scu] one of the paired antero-lateral parts of the cephalic capsule, bearing compound eyes or ocelli when present, and delimited mesally by the antennocellar suture. Fig. 3. Syn.: eye area/areas, ocellary area/areas, ocellary field/fields, ocular area/areas

**compound eye**/eyes: [Scu] faceted vision organ, composed of similar units known as ommatidia. Fig. 3

**ocellus**/ocelli: [Cra, Lit, Sco] simple vision organ, appearing as a single convex lens. Fig. 5

**posterior ocellus**/ocelli: [Lit] the most posterior ocellus on each side of the head. Fig. 5. Crabill 1961a: 132. Syn.: major ocellus/ocelli, principal ocellus/ocelli, terminal ocellus/ocelli

**seriate ocellus**/ocelli: [Lit] one of the ocelli other than the posterior ocellus. Fig. 5. Crabill 1961a: 132. Syn.: minor ocellus/ocelli

**ocellar series**/series: [Lit] one of the sub-horizontal rows in which the seriate ocelli can be arranged. Syn.: ocellar row/rows

**posterosuperior ocellus**/ocelli: [Lit] the most posterior ocellus of the most dorsal row of seriate ocelli. Fig. 5

**Tömösváry’s organ**/organs: [Cra, Lit, Scu] hygroreceptor sensory organ at the side of the head. Fig. 5. Syn.: organ/organs of Tömösváry, postantennal organ/organs, Tömösváry organ/organs

(cephalic) **paramedian suture**/sutures: [Sco] one of the paired paramedian sutures on the cephalic plate. Fig. 6

(cephalic) **paramedian sulcus**/sulci: [Geo, Lit] one of the paired paramedian sulci on the posterior part of the cephalic plate. Fig. 4. Syn.: paired posterior depression/depressions.

(cephalic) **marginal ridge**: [Lit, Sco] narrow ridge along the lateral and posterior margins of the dorsal side of the cephalic capsule. Fig. 1. Crabill 1961a: 131; Lewis et al. 2005: 7. Syn.: limbus, marginal bulge, marginal rim

(cephalic) **marginal sulcus**/sulci: [Lit, Sco] sulcus between the marginal ridge and the remaining part of the dorsal side of the cephalic capsule. Fig. 1

**lateral marginal interruption**/interruptions (of cephalic plate): [Lit] notch on the lateral margins of the cephalic plate. Eason 1964: 179. Syn.: disjuncture/disjunctures of limbus, lateral termination/terminations of marginal ridge

(cephalic) **basal plate**/plates: [Sco] one of the paired sclerites at the posterior corners of the cephalic plate. Fig. 6

**cephalic pleurite**/pleurites: [Cra, Dev, Geo, Lit, Sco] one of the pleurites lateral to the clypeolabrum. Fig. 7. Syn.: bucca/buccae, cephalic pleura/pleurae, cephalic pleuron/pleura

**transverse suture**/sutures (of cephalic pleurite): [Geo] transverse suture on the cephalic pleurite. Fig. 7. Crabill 1960b: 189. Syn.: buccal suture/sutures, transbuccal suture/sutures

**stilus**/stili: [Geo] sclerotised ridge on the mesal margin of the cephalic pleurite. Fig. 8. Crabill 1959a: 192; 1964a: 168; 1970: 235. Syn.: buccal margin/margins

**anterior incision**/incisions (of stilus): [Geo] notch on the mesal side of the stilus. Fig. 8. Crabill 1959a: 192

**Figures 9–16.**
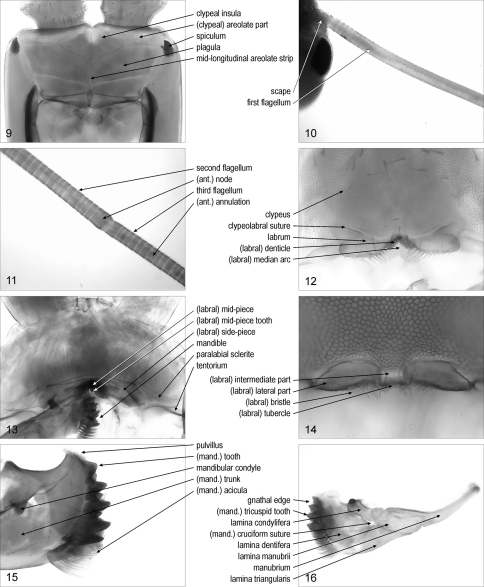
**9** anterior part of cephalic capsule, without maxillary complex and mandibles, ventral, Mecistocephalus togensis **10** basal part of right antenna, dorsal, Scutigera coleoptrata **11** intermediate part of the right antenna, dorsal, Scutigera coleoptrata **12** anterior part of cephalic capsule, without maxillary complex and mandibles, ventral, Pectiniunguis ducalis **13** anterior part of the cephalic capsule, without maxillary complex and left mandible, ventral, Scolopendra oraniensis **14** labrum, ventral, Ribautia centralis **15** distal part of right mandible, antero-dorsal, Lithobius dentatus **16** left mandible, antero-dorsal, Scolopendra oraniensis. Abbreviations: ant., antennal; mand., mandibular.

**spiculum**/spicula: [Geo] sclerotised, pointed projection on the anterior part of the cephalic pleurite. Fig. 9. Crabill 1959a: 192; 1964a: 168; 1970: 236

**maxillary complex**: whole of first and second maxillae

### antenna

**antenna**/antennae: one of the paired most anterior appendages on the head. Fig. 1

(antennal) **article**/articles: one of the rigid sectors along the antenna. Fig. 1. Syn.: (antennal) annulus/annuli, (antennal) joint/joints, (antennal) segment/segments, antennomere/antennomeres

**scape**/scapes: [Scu] set of the two most basal antennal articles. Fig. 10

(antennal) **annulation**/annulations: [Scu] short antennal article. Fig. 11. Syn.: (antennal) article/articles

**flagellum**/flagella: [Scu] one of the sections along the antenna composed of annulations. Figs 10–11. Syn.: duploflagellum/duploflagella

(antennal) **node**/nodes: [Scu] elongate antennal article between two flagella along the antenna. Fig. 11

**first flagellum**/flagella: [Scu] the most basal flagellum along the antenna. Fig. 10. Syn.: flagellum/flagella primum/prima, first division/divisions of antenna/antennae

**second flagellum**/flagella: [Scu] the second flagellum along the antenna. Fig. 11. Syn.: flagellum/flagella secundum/secunda, second division/divisions of antenna/antennae

**third flagellum**/flagella: [Scu] the third flagellum along the antenna. Fig. 11. Syn.: flagellum/flagella tertium/tertia, third division/divisions of antenna/antennae

**shaft organ**/organs: [Scu] sensory organ on the first antennal article

### clypeus and labrum

**clypeolabrum**: antero-ventral part of the cephalic capsule, posterior to the antennae and between the cephalic pleurites. Fig. 7

**clypeus**: sclerite on the antero-ventral part of the cephalic capsule, to the exclusion of the labrum. Fig. 12

**paraclypeal suture**/sutures: one of the lateral margins of the clypeus. Fig. 7. Crabill 1959a: 192; 1959b: 173; 1960b: 189. Syn.: clypeal suture/sutures

**scute**/scutes: area on the cuticle, corresponding to the external face of a single epithelial cell. Fig. 7. Syn.: cuticular polygon/polygons

(clypeal) **areolate part**: [Geo] anterior part of the clypeus that is evidently areolate. Fig. 9. Syn.: areolate clypeus

**plagula**/plagulae: [Geo] one of the non-areolate areas on the posterior part of the clypeus. Fig. 9. Crabill 1959a: 192; 1959b: 173; 1964a: 168; 1970: 235. Syn.: (clypeal or prelabral) consolidated area/areas, (clypeal or prelabral) non-areolate field/fields, (clypeal or prelabral) non-areolate part/parts, posterior clypeus

**mid-longitudinal areolate strip**: [Geo] mid-longitudinal areolate band separating two paired plagulae. Fig. 9. Syn.: mid-longitudinal areolate stripe

**clypeal insula**/insulae: [Geo] non-areolate area inside the areolate part of the clypeus. Fig. 9

**clypeal area**/areas: [Geo] small, subcircular, median area on the areolate part of the clypeus, with distinctly finer or indistinct areolation. Fig. 7. Syn.: anteroclypeal area/areas, clypeal spot/spots, (clypeal or anterocentral) fenestra/fenestrae

**clypeolabral suture**: suture between clypeus and labrum. Fig. 12

**labrum**: posterior part of the clypeolabrum, sometimes delimited from the clypeus by a suture. Fig. 12. Syn.: upper lip

(labral) **mid-piece**: median sclerite of the labrum. Fig. 13. Syn.: (labral) median piece, (labral) middle piece

(labral) **intermediate part**: [Geo] median part of the labrum, when not a sclerite distinct from the lateral parts. Fig. 14. Syn.: median labromere, (labral) median piece, (labral) middle piece, (labral) middle portion, (labral) mid-piece

(labral) **mid-piece tooth**: sclerotised tooth on the labral mid-piece. Fig. 13

(labral) **side-piece**/side-pieces: one of the paired lateral sclerites of the labrum. Fig. 13. Syn.: (labral) lateral piece/pieces, (labral) lateral portion/portions

(labral) **lateral part**/parts: [Geo] one of the paired lateral parts of the labrum, when not sclerites distinct from the intermediate part. Fig. 14. Syn.: (labral) lateral portion/portions, (labral) side-piece/side-pieces

(labral) **ala**/alae: [Geo] one of the two sclerites composing the labral side-piece. Fig. 8

(labral) **anterior ala**/alae: [Geo] the anterior of the two sclerites composing the labral side-piece. Fig. 8

(labral) **posterior ala**/alae: [Geo] the posterior of the two sclerites composing the labral side-piece. Fig. 8

(labral) **transverse thickened line**/lines: [Geo] sclerotised ridge between the anterior and posterior ala of the labral side-piece. Fig. 8

(labral) **median arc**: [Geo] concave posterior margin of the labral intermediate part. Fig. 12. Syn.: labral arch

(labral) **bristle**/bristles: hair-like, sometimes branching, projection along the posterior margin of the labrum. Fig. 14. Syn.: (labral) branching bristle/bristles, (labral) filament/filaments, (labral) (branched) fimbria/fimbriae, (labral) hair/hairs

(labral) **denticle**/denticles: [Geo] subtriangular, flat projection along the posterior margin of the labrum. Fig. 12. Syn.: (labral) tooth/teeth

(labral) **tubercle**/tubercles: [Geo] subconical, stout projection along the posterior margin of the labrum. Fig. 14. Syn.: (labral) tooth/teeth

**paralabial sclerite**/sclerites: [Lit, Sco] one of the paired sclerites posterior to the clypeus and lateral to the labral side-pieces. Fig. 13. Syn.: coclypeus/coclypei

**tentorium**/tentoria: Y-shaped sclerite whose three limbs are attached to the labral lateral parts, the cephalic pleurite, and the mandibular condyle, respectively. Fig. 13. Syn.: (labral or mandibular) fulcrum/fulcra, (labral or labial) fultura/fulturae, tentorial complex/complexes

### mandible

**mandible**/mandibles: one appendage of the first pair of the mouth-parts. Fig. 13

**mandibular condyle**/condyles: condyle of the mandible serving the articulation with the tentorium. Fig. 15

**gnathal edge**/edges: distal margin of the mandible. Fig. 16. Syn.: apical ridge/ridges, gnathal lobe/lobes, molar edge/edges

**manubrium**/manubria: slender projection of the mandible opposite to the gnathal edge with respect to the mandibular condyle. Fig. 16. Crabill 1960a: 15. Syn.: shank/shanks

(mandibular) **trunk**/trunks: main part of the mandible, to the exclusion of the manubrium and the gnathal edge. Fig. 15. Syn.: (mandibular) body/bodies, (mandibular) corpus/corpora, (mandibular) shaft/shafts

(mandibular) **cruciform suture**/sutures: [Sco] pair of crossed sutures on the mandibular trunk. Fig. 16. Syn.: cruciform fissure/fissures

**lamina**/laminae **manubrii**: [Sco] part of the mandible between manubrium and cruciform suture. Fig. 16. Crabill 1960a: 15

**lamina**/laminae **triangularis**/triangulares: [Sco] part of the mandible between the lamina manubrii and the lamina dentifera, opposite to the lamina condylifera with respect to the cruciform suture. Fig. 16. Crabill 1960a: 15

**lamina**/laminae **dentifera**/dentiferae: [Sco] part of the mandible between apical ridge and cruciform suture. Fig. 16. Crabill 1960a: 15

**lamina**/laminae **condylifera**/condyliferae: [Sco] part of the mandible between the lamina manubrii and the lamina dentifera, including the mandibular condyle and opposite to the lamina triangularis with respect to the cruciform suture. Fig. 16. Crabill 1960a: 15

**molar plate**/plates: [Scu] sclerotised, flat area on the gnathal edge

**pulvillus**/pulvilli: array of dense short scales on the dorsal end of the mandibular gnathal edge. Fig. 15. Syn.: furry pad/pads, Haarpolster

(mandibular) **acicula**/aciculae: one of the slender long projections on the ventral end of the mandibular gnathal edge. Fig. 15. Edgecombe 2001: 203. Syn.: sickle bristle/bristles, sickle-shaped bristle/bristles

**Figures 17–24.**
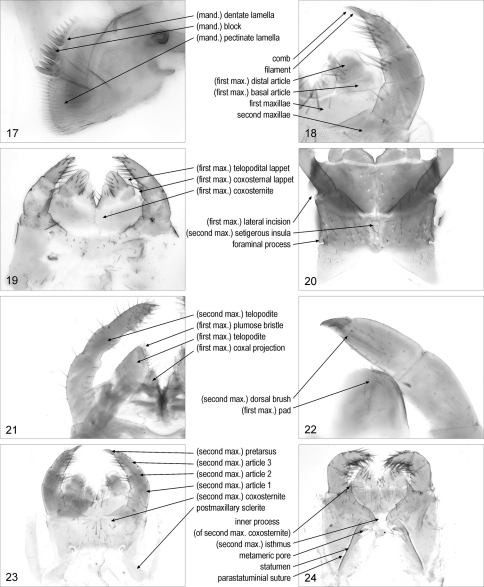
**17** distal part of right mandible, posterior, Pectiniunguis ducalis **18** left part of maxillary complex, ventral, Pectiniunguis ducalis **19** maxillary complex, ventral, Geophilus carpophagus **20** maxillary complex, ventral, Mecistocephalus togensis **21** right part of maxillary complex, ventral, Lithobius dentatus **22** anterior right part of maxillary complex, dorsal, Scolopendra oraniensis **23** maxillary complex, ventral, Pectiniunguis ducalis **24** maxillary complex, ventral, Ribautia centralis. Abbreviations: mand., mandibular; max., maxillary.

**pinnule**/pinnules (of acicula): one of the branches of a mandibular acicula

(mandibular) **branching bristle**/bristles: [Lit] hair-like, branching projection fringing the mandibular teeth and aciculae

(mandibular) **accessory denticle**/denticles: [Lit, Sco] one of the denticles arranged in rows on the mandibular teeth

(mandibular) **lamella**/lamellae: one of the flat projections on the gnathal edge of the mandible. Fig. 17. Syn.: (mandibular) lamina/laminae

(mandibular) **dentate lamella**/lamellae: mandibular lamella bearing teeth. Fig. 17. Syn.: dentate lamina/laminae, dentate plate/plates, lamella/lamellae dentata/dentatae

(mandibular) **tooth**/teeth: sclerotised, large, subconical, projection on a mandibular dentate lamella. Fig. 15

(mandibular) **tricuspid tooth**/teeth: tooth with three tips on a dentate lamella. Fig. 16

(mandibular) **block**/blocks: one of the sclerotised distinct parts of a dentate lamella, each bearing one or more teeth. Fig. 17

(mandibular) **pectinate lamella**/lamellae: [Geo, Sco] mandibular lamella bearing poorly sclerotised, subcylindrical, slender projections. Fig. 17. Syn.: pectinate lamina/laminae

(mandibular) **basal tooth**/teeth: [Geo] subconical projection at the base of the first mandibular lamella

### first maxillae

**first maxillae** {plural only}: pair of appendages and associated basal sclerites between the mandibles and the second maxillae. Fig. 18. Syn.: first maxilla {singular}, maxillae I {plural}

(first maxillary) **sternite**: most basal part of the coxosternite, associated with the first maxillae

(first maxillary) **coxa**/coxae: part of the coxosternite corresponding to a coxa, of the first maxillae

(first maxillary) **coxosternite**: entire sclerite corresponding to sternite and coxae of the first maxillae. Fig. 19. Syn.: coxae {plural}, coxites {plural}, coxosterna {singular}, coxosternum, sternum, syncoxite, syncoxosternum

(first maxillary) **lateral incision**: [Geo] notch on the lateral margin of the first maxillary coxosternite. Fig. 20. Crabil 1959a: 192

(first maxillary) **coxal projection**/projections: one of the paired projections on the anterior margin of the first maxillary coxosternite, mesal to the telopodites. Fig. 21. Syn.: coxal process/processes, inner branch/branches, inner lobe/lobes, medial lobe/lobes, medial projection/projections

(first maxillary) **telopodite**/telopodites: one of the paired projections, usually articulated at the base, on the anterior margin of the first maxillary coxosternite, lateral to the coxal projections. Fig. 21. Syn.: outer branch/branches, outer lobe/lobes, palp/palps, palpus/palpi

(first maxillary) **basal article**/articles: the most basal article of the first maxillary telopodite. Fig. 18. Syn.: femoroid/femoroids

(first maxillary) **distal article**/articles: the most distal article of the first maxillary telopodite. Fig. 18. Syn.: tibio-tarsus/tibio-tarsi

(first maxillary) **plumose bristle**/bristles: [Lit] one of the feather-like projections on the distal article of the first maxillary telopodite. Fig. 21

(first maxillary) **pad**/pads: [Sco] array of short, dense projections on the distal article of the first maxillary telopodite. Fig. 22

(first maxillary) **lappet**/lappets: [Geo] projection on the lateral margin of the first maxillary coxosternite or telopodite. Fig. 19. Syn.: external sensory lappet/lappets, (lateral or maxillary) palp/palps, palpal process/processes, (lateral or maxillary) palpus/palpi

(first maxillary) **coxosternal lappet**/lappets: [Geo] lappet on the first maxillary coxosternite. Fig. 19. Syn.: coxal palpus/palpi, syncoxal lobe/lobes, syncoxital lappet/lappets

(first maxillary) **telopodital lappet**/lappets: [Geo] lappet on the basal article of the first maxillary telopodite. Fig. 19. Syn.: femural palpus/palpi

### second maxillae

**second maxillae** {plural only}: pair of appendages and associated basal sclerite/s, posterior to the first maxillae. Fig. 18. Syn.: labium, maxillae II {plural}, second maxilla {singular}

(second maxillary) **coxosternite**: entire sclerite corresponding to sternite and coxae of the second maxillae. Fig. 23. Syn.: (second maxillary) coxosterna {singular}, (second maxillary) coxosternum

(second maxillary) **isthmus**: median part of the second maxillary coxosternite. Fig. 24

(second maxillary) **setigerous insula**/insulae: [Geo] one of the nonareolate areas, bearing setae, inside the areolate part of the second maxillary coxosternite. Fig. 20

**metameric pore**/pores: one of the paired pores of the maxillary glands on the second maxillary coxosternite. Fig. 24. Syn.: salivary pore/pores

**foraminal process**/processes: [Geo] marginal projection of the second maxillary coxosternite surrounding the metameric pore. Fig. 20

**statumen**/statuminia: [Geo] sclerotised elongated ridge mesal to the metameric pore. Fig. 24. Crabill 1955: 222; 1960b: 194. Syn.: (second maxillary) pleurocoxal line/lines, (second maxillary) pleurosternal suture/sutures

**parastatuminial suture**/sutures: [Geo] suture along the statumen. Fig. 24. Crabill 1964b: 39

**circumforaminal ring**/rings: [Geo] sclerotised ring partially surrounding the metameric pore

**inner process**/processes (of second maxillary coxosternite): [Geo] one of the paired projections on the anterior margin of the second maxillary coxosternite, mesal to the telopodites. Fig. 24. Syn.: (second maxillary) mesodistal process/processes

**Figures 25–32.**
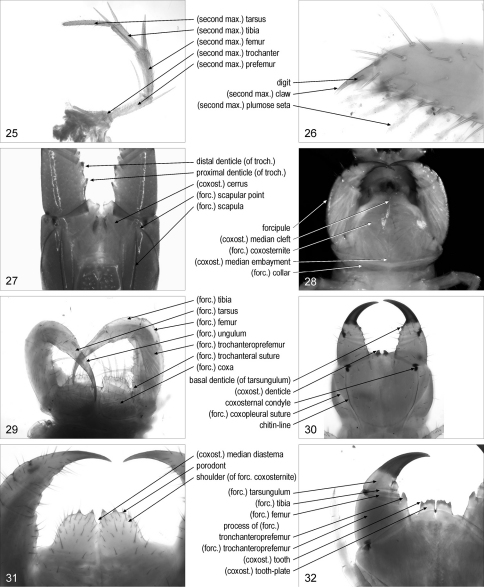
**25** left part of second maxillae, ventral, Scutigera coleoptrata **26** distal part of second maxillary right telopodite, dorsal, Lithobius dentatus **27** forcipular segment, dorsal, Mecistocephalus togensis **28** anterior part of body, ventral, Lamyctes emarginatus **29** forcipular segment, ventral, Scutigera coleoptrata **30** forcipular segment, ventral, Clinopodes trebevicensis **31** anterior part of forcipular segment, ventral, Lithobius dentatus **32** right part of forcipular segment, ventral, Scolopendra oraniensis. Abbreviations: coxost., coxosternal; forc., forcipular; max., maxillary; troch., trochanteroprefemur.

**postmaxillary sclerite**/sclerites: [Cra, Geo, Lit] one of the paired sclerites adjacent to the posterior corners of the second maxillary coxosternite. Fig. 23. Crabill 1960b: 189.

(second maxillary) **telopodite**/telopodites: part of the appendage of the second maxillae, distal to the most basal articulation. Fig. 21. Syn.: palp/palps, palpus/palpi, telopod/telopods

(second maxillary) **trochanter**/trochanters: [Scu] first article of the second maxillary telopodite. Fig. 25

(second maxillary) **prefemur**/prefemora: [Scu] second article of the second maxillary telopodite. Fig. 25

(second maxillary) **femur**/femora: [Scu] third article of the second maxillary telopodite. Fig. 25

(second maxillary) **tibia**/tibiae: [Scu] fourth article of the second maxillary telopodite. Fig. 25

(second maxillary) **tarsus**/tarsi: [Scu] fifth article of the second maxillary telopodite. Fig. 25

(second maxillary) **article**/articles **1**: [Cra, Geo, Lit, Sco] first article of the second maxillary telopodite. Fig. 23. Lewis et al. 2005: 2, 3. Syn.: basal article/articles, femoroid/femoroids, first article/articles, telomere/telomeres 1

(second maxillary) **article**/articles **2**: [Cra, Geo, Lit, Sco] second article of the second maxillary telopodite. Fig. 23. Lewis et al. 2005: 2, 3. Syn.: second article/articles, second joint/joints, telomere/telomeres 2, tibia/tibiae

(second maxillary) **article**/articles **3**: [Cra, Geo, Lit, Sco] third article of the second maxillary telopodite. Fig. 23. Lewis et al. 2005: 2, 3. Syn.: apical article/articles, tarsus/tarsi, telomere/telomeres 3, terminal joint/joints, third article/articles, ultimate article/articles

(second maxillary) **plumose seta**/setae: [Lit] one of the setae with apical branches, on article 3 of the second maxillary telopodite. Fig. 26

(second maxillary) **dorsal brush**/brushes: [Cra, Sco] longitudinal row of hairs on article 3 of the second maxillary telopodite. Fig. 22. Lewis et al. 2005: 2, 3. Syn.: palisade/palisades of capitate hairs

(second maxillary) **pretarsus**/pretarsi: [Cra, Geo, Lit, Sco] terminal element articulated to the most distal article of the second maxillary telopodite. Fig. 23. Syn.: praetarsus/praetarsi

(second maxillary) **claw**/claws: [Cra, Geo, Lit, Sco] second maxillary pretarsus in shape of a claw. Fig. 26. Syn.: apical claw/claws, pretarsal claw/claws, terminal claw/claws

**digit**/digits (of second maxillary claw): [Cra, Lit] one of the short projections on the second maxillary claw. Fig. 26

**comb**/combs (of second maxillary claw): [Geo, Sco] row of projections along the margin of the second maxillary claw. Fig. 18. Syn.: comb/combs of teeth

**filament**/filaments (of second maxillary claw): [Geo, Sco] one of the slender projections of the comb of the second maxillary claw. Fig. 18

### forcipular segment

**forcipular segment**: segment bearing the forcipules. Fig. 2. Syn.: maxillipede segment, prehensorial segment

**forcipular pretergite**: [Geo, Sco] short sclerite anterior to the forcipular tergite. Fig. 2. Syn.: lamina basalis, prebasal plate

**forcipular tergite**: main tergite of the forcipular segment. Fig. 2. Syn.: basal plate

**forcipular pleurite**/pleurites: lateral sclerite of the forcipular segment. Fig. 2. Syn.: (forcipular or maxillipede) pleuron/pleura, (forcipular or maxillipede) pleura/pleurae

(forcipular) **scapula**/scapulae: [Geo] dorsal ridge of the forcipular pleurite. Fig. 27

(forcipular) **scapular point**/points: [Geo] projecting anterior tip of the forcipular scapula. Fig. 27. Crabill 1970: 237. Syn.: scapular projection/projections

(forcipular) **collar**: [Lit] ventral transversal bridge connecting the forcipular pleurites. Fig. 28. Syn.: (maxillipede) (pleural) collar

(forcipular) **coxa**/coxae: [Scu] one of the paired sclerites basal to the forcipules, bearing spine-bristles on the anterior margin. Fig. 29. Syn.: (forcipular) coxite/coxites

(forcipular) **coxosternite**: [Cra, Dev, Geo, Lit, Sco] entire sclerite corresponding to sternite and coxae of the forcipular segment. Fig. 28. Syn.: (maxillipede) coxosternite, (forcipular or maxillipede) coxosternum, (prehensorial) pre-sternum, (prehensorial) prosternum

(forcipular) **coxopleural suture**/sutures: suture between the forcipular pleurite and the forcipular coxae or coxosternite. Fig. 30. Syn.: pleuroprosternal suture/sutures

**coxosternal condyle**/condyles: condyle of the forcipular coxa or coxosternite serving the articulation with the trochanteroprefemur. Fig. 30. Syn.: (coxofemoral or prehensorial) condyle/condyles

(coxosternal) **cerrus**/cerri: [Geo] one of the paired groups of setae on the dorsal side of the forcipular coxosternite. Fig. 27. Crabill 1970: 236

(coxosternal) **condylar process**/processes: [Geo] one of the paired projections of the forcipular coxosternite, close to the dorsal coxosternal condyles

**shoulder**/shoulders (of forcipular coxosternite): [Lit] one of the paired obtuse projections on the anterior margin of the forcipular coxosternite. Fig. 31. Syn.: coxal endite/endites, lateral prosternal prominence/prominences

(coxosternal) **median diastema**: [Cra, Dev, Geo, Lit, Sco] median concavity on the anterior margin of the forcipular coxosternite. Fig. 31. Syn.: median interval, median notch, median sinus

(coxosternal) **tooth**/teeth: [Cra, Dev, Lit, Sco] sclerotised, short, subconical projection on the anterior margin of the forcipular coxosternite. Fig. 32. Syn.: (coxosternal or prosternal) (anterior or anterocentral) denticle/denticles, (prosternal or forcipular) tooth/teeth, (marginal) tubercle/tubercles

(coxosternal) **tooth-plate**/tooth-plates: [Cra, Sco] one of the paired sclerotised, flat, teeth-bearing projections on the anterior margin of the forcipular coxosternite. Fig. 32. Lewis et al. 2005: 2, 3. Syn.: dental plate/plates, (coxosternal or prosternal) plate/plates, (coxosternal or prosternal) toothed anterior process/processes

(coxosternal) **denticle**/denticles: [Geo] one of the paired small, subconical projections on the anterior margin of the forcipular coxosternite. Fig. 30. Syn.: (coxosternal) tooth/teeth

**porodont**/porodonts: [Lit] one of the paired large setae usually placed lateral to the forcipular coxosternal teeth. Fig. 31. Crabill 1953: 119. Syn.: accessory spine/spines, ectal spine/spines, ectodont/ectodonts, lateral (prosternal) spine/spines, parodontal spine/spines, pseudoporodont/pseudoporodonts

**porodont node**/nodes: [Lit] basal structure from which the porodont arises

(coxosternal) **median cleft**: [Lit] mid-longitudinal suture on the ventral side of the forcipular coxosternite. Fig. 28. Eason 1964: 165.

**chitin-line**/chitin-lines: [Geo] one of the paired paramedian sclerotised narrow stripes on the ventral side of the forcipular coxosternite. Fig. 30. Crabill 1960a: 15.Syn.: chitinous line/lines, pleurogram/pleurograms, (prosternal) (subcondylic) sclerotic line/lines

(coxosternal) **median embayment**: [Lit, Sco] median notch at the posterior margin of the forcipular coxosternite. Fig. 28. Edgecombe and Koch 2008: 895

**forcipule**/forcipules: telopodite of the forcipular segment. Fig. 28. Lewis 1981: 11. Syn.: forcipula/forcipulae, forcipular telopodite/telopodites, maxilliped/maxillipeds, maxillipede/maxillipedes, poison claw/claws, prehensor/prehensors, prehensorial foot/feet, prehensorial telopodite/telopodites, toxicognath/toxicognaths

(forcipular) **trochanteroprefemur**/trochanteroprefemora: first article of the forcipule. Figs 29, 32. Syn.: article/articles 1, basal article/articles, femoroid/femoroids, femuroid/femuroids, first article/articles

(forcipular) **trochanteral suture**/sutures: trace of suture on the forcipular trochanteroprefemur. Fig. 29

(forcipular) **intermediate article**/articles: one of the second and third articles of the forcipule. Syn.: intercalary article/articles, intermediate joint/joints

(forcipular) **femur**/femora: second article of the forcipule. Figs 29, 32. Syn.: article/articles 2, femoroid/femoroids, femuroid/femuroids, second article/articles, second joint/joints, tibia/tibiae

(forcipular) **tibia**/tibiae: third article of the forcipule. Figs 29, 32. Syn.: article/articles 3, tarsus/tarsi, third article/articles, third joint/joints, tibioid/tibioids

(forcipular) **tarsus**/tarsi: [Scu] fourth article of the forcipule. Fig. 29(forcipular)

**ungulum**/ungula: [Scu] terminal articulated element of the forcipule. Fig. 29. Syn.: pretarsus/pretarsi, ungula/ungulae

(forcipular) **tarsungulum**/tarsungula: [Cra, Dev, Geo, Lit, Sco] ultimate article of the forcipule. Fig. 32. Lewis et al. 2005: 2. Syn.: article/articles 4, claw/claws, metatarsus/metatarsi, poison claw/claws, pretarsus/pretarsi, tarsungula/tarsungulae

**process**/processes **of** (forcipular) **tronchanteroprefemur**: [Cra, Sco] large projection on the mesal side of the forcipular trochanteroprefemur. Fig. 32. Lewis et al. 2005: 2, 3. Syn.: (tronchanteroprefemoral) (inner spinous) process/processes, process/processes of femoroid, (tronchanteroprefemoral) (inner or median) tooth/teeth

(forcipular) **denticle**/denticles: [Geo] small subconical projection on the mesal side of the forcipule. Figs 27, 30. Syn.: node/nodes, nodule/nodules, tooth/teeth

**proximal denticle**/denticles (of trochanteroprefemur): [Geo] the most basal of two denticles along the mesal side of the forcipular trochanteroprefemur. Fig. 27. Syn.: basal denticle/denticles (of trochanteroprefemur)

**distal denticle**/denticles (of trochanteroprefemur): [Geo] the most distal of two denticles along the mesal side of the forcipular trochanteroprefemur. Fig. 27

**basal denticle**/denticles (of tarsungulum): [Geo] denticle at the base of the forcipular tarsungulum. Fig. 30. Syn.: basal tooth/teeth, (basal) node/nodes

(forcipular) **spine comb**/combs: [Scu] row of spines on the forcipular tarsus

### leg-bearing segment

**Figures 33–40.**
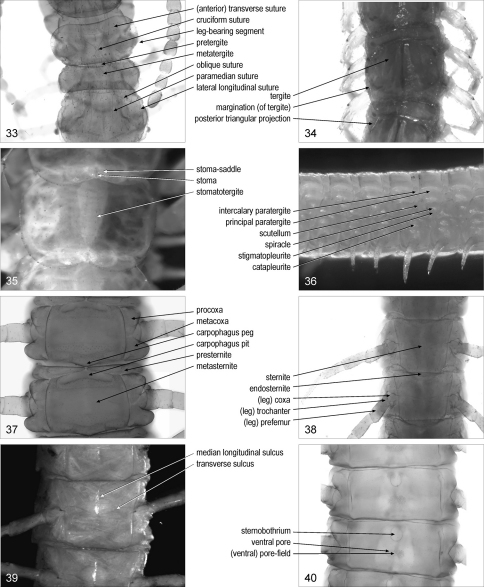
**33** anterior part of trunk, dorsal, Cryptops anomalans **34** intermediate part of trunk, dorsal, Lithobius dentatus **35** intermediate part of trunk, dorsal, Scutigera coleoptrata **36** intermediate part of trunk, left, Himantarium gabrielis **37** intermediate part of trunk, ventral, Clinopodes trebevicensis **38** intermediate part of trunk, ventral, Cryptops parisi **39** intermediate part of trunk, ventral, Cryptops punicus **40** intermediate part of trunk, ventral, Bothriogaster signata.

**leg-bearing segment**/segments: segment of the trunk bearing paired walking appendages. Fig. 33. Syn.: pedal segment/segments, pedigerous (post-maxillipede) segment/segments, trunk-segment/trunk-segments

**tergite**/tergites (of leg-bearing segment): sclerite on the dorsal side of a leg-bearing segment. Fig. 34. Syn.: dorsal plate/plates, dorsal shield/shields, (dorsal) scutum/scuta, tergal plate/plates, tergum/terga {inappropriate use; see, e.g., Snodgrass 1935}

**stomatotergite**/stomatotergites: [Scu] tergite bearing a stoma. Fig. 35

**stoma**/stomata: [Scu] elongate opening of the respiratory organs on the posteromedian part of a stomatotergite. Fig. 35. Syn.: spiracle/spiracles, stigma/stigmata

**stoma-saddle**/stoma-saddles: [Scu] domed region of the stomatotergite surrounding the stoma. Fig. 35. Syn.: saddle/saddles

**margination**/marginations (of tergite): [Lit, Sco] marginal ridge on a tergite. Fig. 34. Lewis et al. 2005: 2. Syn.: border/borders, marginal ridge/ridges

**posterior triangular projection**/projections (of tergite): [Lit] angulated projection on each posterior corner of a tergite. Fig. 34. Syn.: angulation/angulations, posterior production/productions, posterior tergital projection/projections

**pretergite**/pretergites: [Geo, Sco] anterior sclerite of the two dorsal sclerites of a leg-bearing segment. Fig. 33. Syn.: intercalary tergite/tergites, intertergite/intertergites, prescutum/prescuta, pretergum/preterga, protergite/protergites

**metatergite**/metatergites: [Geo, Sco] posterior sclerite of the two dorsal sclerites of a leg-bearing segment. Fig. 33. Syn.: tergite/tergites

**paramedian sulcus**/sulci **or** **suture**/sutures (of tergite): [Geo, Sco] one of the paired paramedian longitudinal sutures or sulci on a tergite. Fig. 33. Lewis et al. 2005: 2, 3, 5. Syn.: paramedian groove/grooves, paramedian longitudinal sulcus/sulci or suture/sutures

(anterior) **transverse sulcus**/sulci **or** **suture**/sutures (of tergite): [Sco] transverse suture or sulcus on the first trunk tergite. Fig. 33. Lewis et al. 2005: 2, 3. Syn.: (procurved) cervical groove/grooves, (anterior) cervical sulcus/sulci or suture/sutures, semi-lunar sulcus/sulci, T1 ring suture/sutures, transversal suture/sutures, transverse collar sulcus/sulci, (procurved) transverse groove/grooves

**cruciform suture**/sutures (of tergite): [Sco] pair of crossed sutures on the first trunk tergite. Fig. 33

**oblique suture**/sutures (of tergite): [Sco] one of the paired oblique sutures on some anterior trunk tergites. Fig. 33. Lewis et al. 2005: 5. Syn.: arcuate suture/sutures, curved lateral sulcus/sulci

**lateral longitudinal suture**/sutures (of tergite): [Sco] one of the paired longitudinal sutures close to the lateral margins of a tergite. Fig. 33. Lewis et al. 2005: 5

**lateral crescentic sulcus**/sulci (of tergite): [Sco] one of the paired curved, sublongitudinal sulci on a tergite. Lewis et al. 2005: 5

**eupleurium**/eupleuria: the whole of pleurites on a side of a leg-bearing segment. Eason 1964: 277. Syn.: pleuron/pleura

**pleural membrane**/membranes: arthrodial membrane between pleurites

**paratergite**/paratergites: [Geo] pleurite contiguous or almost contiguous to a tergite. Fig. 36

**intercalary paratergite**/paratergites: [Geo] paratergite lateral to a pretergite. Fig. 36. Crabill 1960c: 93. Syn.: parapretergite/parapretergites, preparatergite/preparatergites, secondary paratergite/paratergites, suprascutellum/suprascutella

**primary intercalary paratergite**/paratergites: [Geo] the most mesal of two intercalary paratergites. Fig. 36. Syn.: primary suprascutellum/suprascutella

**secondary intercalary paratergite**/paratergites: [Geo] the most distal of two intercalary paratergites. Fig. 36. Syn.: secondary suprascutellum/suprascutella

**principal paratergite**/paratergites: [Geo] paratergite lateral to a metatergite. Fig. 36. Crabill 1960c: 93. Syn.: major paratergite/paratergites, primary paratergite/paratergites

**scutellum**/scutella: [Geo, Sco] pleurite in antero-ventral position with respect to the stigmatopleurite. Fig. 36. Syn.: intercalary pleurite/pleurites, prescutellum/prescutella

**spiracle**/spiracles: [Cra, Dev, Geo, Lit, Sco] one of the paired openings of the tracheae on the lateral sides of a leg-bearing segment. Fig. 36. Syn.: stigma/stigmata

**stigmatopleurite**/stigmatopleurites: [Cra, Dev, Geo, Lit, Sco] pleurite bearing a spiracle. Fig. 36. Syn.: spiracle-bearing pleurite/pleurites, spiraculiferous plate/plates, stigma-bearing pleurite/pleurites, stigmopleurite/stigmopleurites

**catapleurite**/catapleurites: pleurite between the scutellum and the coxa. Fig. 36. Syn.: catopleure/catopleures, katopleure/katopleures

**eucoxa**/eucoxae **superior**/superiores: [Lit] pleurite dorsal to the coxa

**eucoxa**/eucoxae **inferior**/inferiores: [Lit] pleurite ventral to the coxa

**pleurocoxa**/pleurocoxae: [Geo] pleurite between coxa and metacoxa

**subcoxa**/subcoxae: [Cra, Dev, Geo, Lit, Sco] pleurite anterior or posterior to the coxa. Syn.: subcoxal pleurite/pleurites

**procoxa**/procoxae: [Cra, Dev, Geo, Lit, Sco] subcoxa anterior to the coxa. Fig. 37. Syn.: anterior subcoxal plate/plates, precoxa/precoxae, procoxal pleurite/pleurites, prosubcoxa/prosubcoxae

**metacoxa**/metacoxae: [Cra, Dev, Geo, Lit, Sco] subcoxa posterior to the coxa. Fig. 37. Syn.: metacoxal pleurite/pleurites, metasubcoxa/metasubcoxae

**sternite**/sternites (of leg-bearing segment): sclerite on the ventral side of a leg-bearing segment. Fig. 38. Syn.: sternum/sterna {inappropriate use; see, e.g., Snodgrass, 1935}, ventral plate/plates, ventral shield/shields

**presternite**/presternites: [Cra, Dev, Geo, Sco] anterior region of the single sternite of a leg-bearing segment, or anterior sclerite of the two ventral sclerites of a leg-bearing segment. Fig. 37. Syn.: intercalary sternite/sternites, intersternite/intersternites, prosternite/prosternites

**metasternite**/metasternites: [Cra, Dev, Geo, Sco] posterior region of the single sternite of a leg-bearing segment, or posterior sclerite of the two ventral sclerites of a leg-bearing segment. Fig. 37. Syn.: sternite/sternites

**transverse sulcus**/sulci (of sternite): [Sco] transverse sulcus on a sternite. Fig. 39. Lewis et al. 2005: 5

**median longitudinal sulcus**/sulci (of sternite): [Sco] mid-longitudinal sulcus on a sternite. Fig. 39. Lewis et al. 2005: 5. Syn.: median sulcus/sulci, mid-longitudinal sulcus/sulci

**cruciform suture**/sutures (of sternite): [Sco] the pair of transverse and median longitudinal sulci on a sternite. Fig. 39. Syn.: cross furrow/furrows, cross sulcus/sulci, cruciform impression/impressions, cruciform sulcus/sulci

**trigonal suture**/sutures (of sternite): [Sco] pair of crossed sutures on the posterior part of the sternite. Lewis et al. 2005: 5

**endosternite**/endosternites: [Geo, Sco] posterior projection of a sternite, covered by the sternite of the following segment. Fig. 37. Syn.: metasternite/metasternites

**carpophagus peg**/pegs: [Geo] median projection on the posterior margin of a sternite, in the carpophagus-structure. Fig. 37. Crabill 1954: 174. Syn.: paxillus/paxilli

**carpophagus pit**/pits: [Geo] median socket on the anterior margin of a sternite, in the carpophagus-structure. Fig. 37. Crabill 1954: 174. Syn.: carpophagus fossa/fossae, sacculus/sacculi, sternal pit/pits

**carpophagus-structure**/carpophagus-structures: [Geo] whole of a carpophagus peg and the associated carpophagus pit. Fig. 37

**ventral pore**/pores: [Geo] glandular pore on the ventral side of a leg-bearing segment. Fig. 40. Syn.: sternal pore/pores, sternital pore/pores

(ventral) **pore-field**/pore-fields: [Geo] an area of clustered pores on the ventral side of a leg-bearing segment. Fig. 40. Syn.: pore area/areas, pore-group/pore-groups, poriferous area/areas, porigerous area/areas

**sternobothrium**/sternobothria: [Geo] median horseshoe-like pit on the metasternite. Fig. 40

**Figures 41–48.**
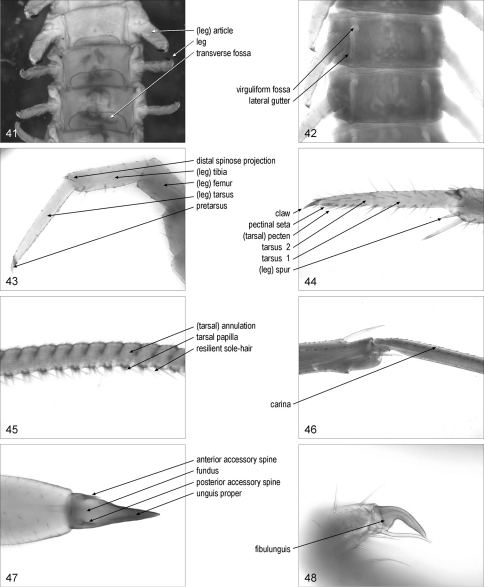
**41** intermediate part of trunk, ventral, Haplophilus souletinus **42** intermediate part of trunk, ventral, Stigmatogaster gracilis **43** right leg, anterior, Lamyctes emarginatus **44** distal part of right leg, antero-ventral, Lithobius dentatus **45** tarsus of left leg, anterior, Scutigera coleoptrata **46** intermediate part of left leg, anterior, Scutigera coleoptrata **47** distal part of left leg, ventral, Scolopendra oraniensis **48** distal part of left leg, anterior, Diphyonyx conjungens

**transverse fossa**/fossae (of sternite): [Geo] transverse, ellipical depression on some trunk sternites. Fig. 41. Eason 1964: 54

**fungiform fovea**/foveae: [Geo] median T-like pit on the metasternite

**virguliform fossa**/fossae: [Geo] comma-like pit at each of the anterior corners of a sternite. Fig. 42. Eason 1964: 284. Syn.: sternal pouch/pouches

**lateral gutter**/gutters (of sternite): [Geo] longitudinal groove along the lateral margin of a sternite. Fig. 42. Eason 1964: 48. Syn.: parasternital cleft/clefts, parasternital fossa/fossae, parasternital pit/pits

### leg

**leg**/legs: one of the paired appendages of the trunk to the exclusion of the forcipules and the gonopods. Fig. 41. Syn.: (ambulatory or locomotory or walking) leg/legs

**cursiped**/cursipeds: [Lit] a leg of the pairs 1–13. Crabill 1961a: 132

**tenaciped**/tenacipeds: [Lit] a leg of the pairs 14–15. Crabill 1961a: 132

(leg) **article**/articles: one of the articulated elements of a leg. Fig. 41. Syn.: podomere/podomeres, (leg) segment/segments

(leg) **coxa**/coxae: the most basal article of a leg. Fig. 38

(leg) **trochanter**/trochanters: small, basalmost article of a telopodite. Fig. 38

(leg) **prefemur**/prefemora: second article of a telopodite. Fig. 38. Syn.: praefemur/praefemora

(leg) **femur**/femora: third article of a telopodite. Fig. 43

(leg) **tibia**/tibiae: fourth article of a telopodite. Fig. 43. Syn.: patellotibia/patellotibiae

(leg) **tarsus**/tarsi: fifth article of a telopodite, when ultimate. Fig. 43

**tarsal article**/articles: one of the articles of a biarticulated region of the leg corresponding to the tarsus. Syn.: tarsalium/tarsalia, tarsomere/tarsomeres

**tarsus**/tarsi **1**: the basal article of two tarsal articles. Fig. 44. Lewis et al. 2005: 2. Syn.: basitarsus/basitarsi, first division/divisions of tarsus/tarsi, first tarsal article/articles, first tarsal joint/joints, first tarsal segment/segments, first tarsus/tarsi, I tarsus/tarsi, protarsus/protarsi, proximotarsus/proximotarsi, tarsomere/tarsomeres 1, tarsus/tarsi, tarsus/tarsi I

**tarsus**/tarsi **2**: the distal article of two tarsal articles. Fig. 44. Lewis et al. 2005: 2. Syn.: distitarsus/distitarsi, distotarsus/distotarsi, II tarsus/tarsi, metatarsus/metatarsi, pretarsus/pretarsi, second division/divisions of tarsus/tarsi, second tarsal article/articles, second tarsal joint/joints, second tarsal segment/segments, second tarsus/tarsi, tarsomere/tarsomeres 2, tarsus/tarsi II, telotarsus/telotarsi

(tarsal) **annulation**/annulations: [Lit, Sco, Scu] each part of a tarsal article, between two contiguous constrictions. Fig. 45. Syn.: annulus/annuli, pseudosegment/pseudosegments, secondary article/articles, tarsale/tarsalia, tarsomere/tarsomeres

**carina**/carinae: [Scu] longitudinal ridge on a leg article. Fig. 46. Edgecombe and Giribet 2006: 509

**tarsal papilla**/papillae: [Scu] relatively short, stout projection with a rounded tip, on the ventral side of tarsus 2. Fig. 45. Edgecombe and Giribet 2006: 509

**resilient sole-hair**/sole-hairs: [Scu] one of the paired hairs thickened at the base, on the ventral side of the leg, originating near the posteromedian margin of each tarsal papilla. Fig. 45. Würmli 1974: 95; Edgecombe and Giribet 2006: 512

(leg) **spur**/spurs: [Lit, Sco] spur on legs. Fig. 44. Syn.: calcar/calcars, (leg or pedal) spine/spines, spiniform seta/setae

**distal spinose projection**/projections (of tibia): [Lit] spinous process at the distal end of the tibia of a leg. Fig. 43. Syn.: tibial spur/spurs

**pectinal seta**/setae: [Lit] one of the decumbent setae arranged in rows along the tarsal articles of legs. Fig. 44. Crabill 1958: 262

(tarsal) **pecten**/pectines: [Lit] row of pectinal setae. Fig. 44. Crabill 1958: 262

**pretarsus**/pretarsi: apical element articulated at the tip of a leg. Fig. 43. Lewis et al. 2005: 2. Syn.: praetarsus/praetarsi, postarsus/postarsi, posttarsus/posttarsi

**claw**/claws: pretarsus in shape of a claw. Fig. 44. Lewis et al. 2005: 2. Syn.: apical claw/claws, end claw/claws, tarsal claw/claws

**fundus**/fundi (of claw): basal, swollen part of a claw. Fig. 47. Crabill 1961b: 501

**unguis**/ungues **proper**: distal, slender part of a claw. Fig. 47. Syn.: claw/claws proper, main claw/claws, principal claw/claws, unguis/ungues

**accessory spine**/spines: slender, pointed projection at the base of the claw. Fig. 47. Lewis et al. 2005: 2, 3. Syn.: accessory claw/claws, accessory seta/setae, accessory spur/spurs, basal bristle/bristles, basal spine/spines, basal spur/spurs, claw spine/spines, parunguis/parungues, sensory spine/spines, sensory spur/spurs

**anterior accessory spine**/spines: the anterior accessory spine of two of a claw. Fig. 47. Syn.: anterior accessory claw/claws, anterior accessory spur/spurs, anterior parunguis/parungues

**posterior accessory spine**/spines: the posterior accessory spine of two of a claw. Fig. 47. Syn.: posterior accessory claw/claws, posterior accessory spur/spurs, posterior parunguis/parungues

(pretarsal) **posteroventral spine**/spines: [Lit] the largest of a pair of spines emerging on the ventro-posterior side at the base of a claw, close to the posterior accessory spine. Edgecombe 2004: 31; Koch and Edgecombe 2008: 168

(pretarsal) **subsidiary spine**/spines: [Lit] the smallest of a pair of spines emerging on the ventro-posterior side at the base of a claw, close to the posterior accessory spine. Edgecombe 2004: 31

**fibulunguis**/fibulungues: [Geo] pretarsus with a large projection flanking the unguis proper. Fig. 48. Crabill 1969: 38

**Figures 49–56.**
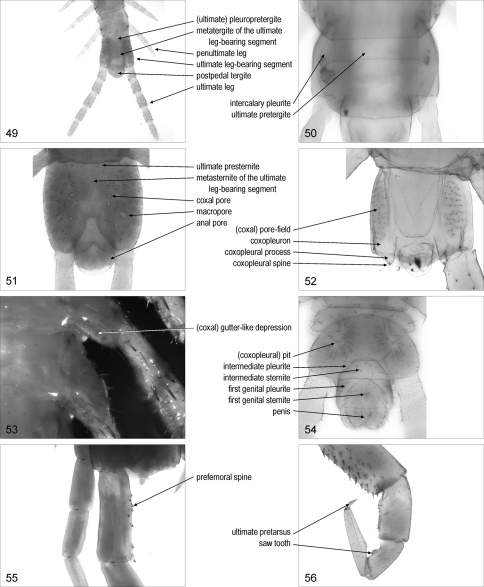
**49** terminal part of trunk, dorsal, Gnathoribautia bonensis **50** ultimate leg-bearing segment, dorsal, Bothriogaster signata **51** terminal part of trunk, ventral, female Dicellophilus carniolensis **52** terminal part of trunk, ventral, Cormocephalus gervaisianus **53** basal part of posterior left legs, ventral, Lithobius dentatus **54** terminal part of trunk, ventral, male Tuoba sydneyensis **55** basal part of ultimate left legs, dorsal, Scolopendra oraniensis **56** ultimate right leg, posterior, Cryptops anomalans.

**penultimate leg**/legs: leg of the penultimate pair. Fig. 49. Syn.: penult leg/legs

### ultimate leg-bearing segment

**ultimate leg-bearing segment**: leg-bearing segment bearing the ultimate pair of legs. Fig. 49. Syn.: last leg-bearing segment, last pediferous segment, last trunk-segment, ultimate pedal segment, ultimate pedigerous segment

**ultimate pretergite**: [Geo] pretergite of the ultimate leg-bearing segment. Fig. 50

**intercalary pleurite**/pleurites: [Geo] pleurite contiguous to the ultimate pretergite. Fig. 50. Syn.: prepleurite/prepleurites

(ultimate) **pleuropretergite**: [Geo] entire sclerite corresponding to the ultimate pretergite and the two intercalary pleurites. Fig. 49

**tergite** (or metatergite) **of the ultimate leg-bearing segment**: main tergite of the ultimate leg-bearing segment. Fig. 49. Syn.: last dorsal plate, last tergite, ultimate tergite

**ultimate presternite**: [Geo] presternite of the ultimate leg-bearing segment. Fig. 51

**sternite** (or metasternite) **of the ultimate leg-bearing segment**: main sternite of the ultimate leg-bearing segment. Fig. 51. Syn.: last sternite, last sternum, last ventral plate, sternite (or metasternite) of the last trunk segment, sternite (or metasternite) of the last leg-bearing segment, ultimate (pedal) sternite

**ultimate leg**/legs: one of the legs of the ultimate pair. Fig. 49. Syn.: anal leg/legs, caudal leg/legs, end leg/legs, last leg/legs, posterior leg/legs, terminal leg/legs

**coxopleuron**/coxopleura: [Geo, Sco] basal element of the ultimate leg, corresponding to coxa and pleurites. Fig. 52. Lewis et al. 2005: 2. Syn.: (anal or last) coxa/coxae, coxopleura/coxopleurae, coxopleurite/coxopleurites, (last) pleura/pleurae, pleurocoxa/pleurocoxae

**coxopleural process**/processes: [Sco] posterior process of the coxopleuron. Fig. 52. Lewis et al. 2005: 2. Syn.: coxal process/processes, process/processes of last coxa/coxae

(coxopleural) **spine**/spines: [Sco] spine on the coxopleuron. Fig. 52. Lewis et al. 2005: 2, 5. Syn.: (coxopleural) spur/spurs

(coxopleural) **side spine**/spines: [Sco] spine on the posterior margin of the coxopleuron mesal to the coxopleural process. Lewis et al. 2005: 5

**coxal pore**/pores: [Cra, Dev, Geo, Lit, Sco] one of the pores of the coxal organs on posterior legs. Fig. 51. Syn.: coxopleural pore/pores, pleural pore/pores

**macropore**/macropores: [Geo] coxal pore that is distinctly larger than the other pores. Fig. 51

(coxal) **gutter-like depression**/depressions: [Lit] depressed area on the coxopleuron containing the openings of the coxal organs. Fig. 53

(coxopleural) **pit**/pits: [Geo] pit on the coxopleuron, containing the openings of the coxal organs. Fig. 54. Crabill 1961a: 133. Syn.: (coxal or coxopleural) crypt/crypts, gland pit/pits, subsurface gland-pit/gland-pits, subsurface pocket/pockets

(coxopleural) **fossa**/fossae: [Geo] longitudinal pouch close to the mesal margin of the coxopleuron, containing the openings of the coxal organs. Syn.: (coxopleural or porigerous) cavity/cavities, (coxopleural or porigerous) fossula/fossulae

(coxal) **pore-field**/pore-fields: [Cra, Dev, Geo, Lit, Sco] part of the surface of the coxa or coxopleuron of the ultimate legs containing the coxal pores. Fig. 52. Syn.: (coxal) cribriform area/areas, (coxal) porose area/areas

**prefemoral spine**/spines: [Sco] spine on the prefemur of ultimate and/or penultimate legs. Fig. 55. Lewis et al. 2005: 3, 5. Syn.: prefemoral dorsal spur/spurs, prefemoral tooth/teeth

**prefemoral** (spinous) **process**/processes: [Sco] process, usually bearing spines, on the prefemur of the ultimate legs. Lewis et al. 2005: 3, 5, 7. Syn.: distomedial prefemoral tubercle/tubercles(prefemoral)

**corner spine**/spines: [Sco] spine on the distal end, on the mesal side, of the prefemur of ultimate legs. Lewis et al. 2005: 3, 5. Syn.: (prefemoral) distomedial spine/spines

**saw tooth**/teeth: [Sco] one of the bluntly pointed projections arranged in rows on the tibia and tarsus 1 of ultimate legs. Fig. 56. Lewis et al. 2005: 7. Syn.: (tibial and tarsal) (serrate) comb/combs, mucro/mucrones, saw-like tooth/teeth

**ultimate pretarsus**/pretarsi: pretarsus of the ultimate leg. Fig. 56

### terminal part of the body

**Figures 57–64.**
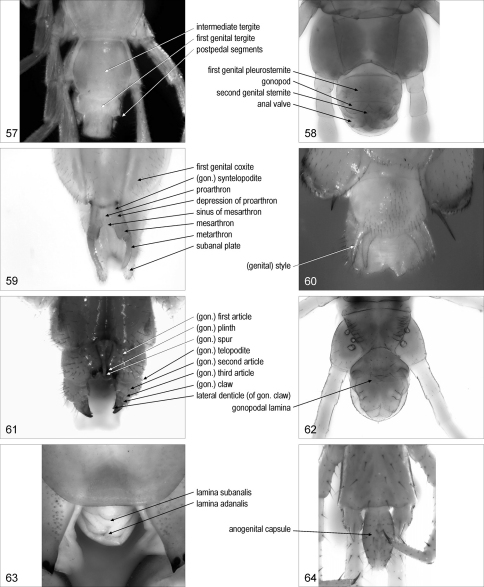
**57** terminal part of trunk, dorsal, Lamyctes emarginatus **58** terminal part of trunk, ventral, female Bothriogaster signata **59** postpedal segments, ventral, female Scutigera coleoptrata **60** postpedal segments, ventral, male Scutigera coleoptrata **61** postpedal segments, ventral, female Lithobius dentatus **62** posterior part of trunk, ventral, female Geophilus carpophagus **63** postpedal segments, ventral, Scolopendra cingulata **64** terminal part of trunk, ventral, Craterostigmus tasmanianus. Abbreviations: gon., gonopodal.

**postpedal segments** {plural only}: part of the trunk posterior to the ultimate leg-bearing segment. Fig. 57. Syn.: terminal segments

**intermediate tergite**: [Lit] tergite posterior to the tergite of the ultimate leg-bearing segment, corresponding to the intermediate sternite. Fig. 57. Eason 1964: 167

**intermediate sternite**: sternite between the sternite or metasternite of the ultimate leg-bearing segment and the first genital sternite. Fig. 54

**intermediate pleurite**/pleurites: [Geo] one of the two pleurites flanking the intermediate sternite. Fig. 54

**first genital tergite**: [Lit] tergite posterior to the intermediate tergite, corresponding to the first genital sternite. Fig. 57

**first genital sternite**: sternite between the intermediate sternite and the second genital sternite, usually associated with gonopods. Fig. 54. Syn.: pregenital sternite, sternite of first genital segment

**first genital pleurite**/pleurites: one of the two pleurites flanking the first genital sternite. Fig. 54. Syn.: pleurite of first genital segment

**first genital pleurosternite**: entire sclerite corresponding to the first genital sternite and the relevant pleurites. Fig. 58

**first genital coxite**/coxites: [Scu] one of the paired sclerites lateral to the first genital sternite. Fig. 59

**second genital sternite**: sternite posterior to the first genital sternite. Fig. 58. Syn.: genital sternite, sternite of second genital segment

**gonopod**/gonopods: one of the paired appendages usually associated with the first or the second genital sternite. Fig. 58. Syn.: genital appendage/appendages

(genital) **style**/styles: [Scu] styliform male gonopod. Fig. 60

**proarthron**: [Scu] basal part of the complex of the paired female gonopods, composed of the basal contiguous parts of the basal articles of the gonopods. Fig. 59

**depression**/depressions **of proarthron**: [Scu] one of the paired depressions on the proarthron. Fig. 59

**mesarthron**: [Scu] median part of the complex of the paired female gonopods, composed of the distal separated parts of the basal articles of the gonopods. Fig. 59

**sinus of mesarthron**: [Scu] concave median posterior margin of the mesarthron. Fig. 59

**metarthron**: [Scu] terminal part of the complex of the paired female gonopods. Fig. 59

(gonopodal) **syntelopodite**: [Scu] coalescent pair of female gonopod telopodites. Fig. 59

(gonopodal) **first article**/articles: [Cra, Dev, Geo, Lit] basal article of the gonopod. Fig. 61. Syn.: (gonopodal) basal article/articles, (gonopodal) coxa/coxae, (gonopodal) coxite/coxites, (gonopodal) segment/segments 1

(gonopodal **telopodite**/telopodites: [Lit] articles of the gonopod other than the first article. Fig. 61

(gonopodal) **second article**/articles: [Cra, Dev, Geo, Lit] second article of the gonopod. Fig. 61. Syn.: (gonopodal) segment/segments 2

(gonopodal) **third article**/articles: [Lit] third article of the gonopod. Fig. 61. Syn.: (gonopodal) segment/segments 3

(gonopodal) **claw**/claws: [Lit] apical claw of the female gonopod. Fig. 61

(gonopodal) **spur**/spurs: [Lit] spur on the female gonopod. Fig. 61. Syn.: (gonopodal) basal spine/spines, (gonopodal) (accessory) denticle/denticles, macroseta/macrosetae

(gonopodal) **plinth**/plinths: [Lit] swollen projection bearing a gonopodal spur. Fig. 61

(gonopodal) **papilla**/papillae: [Lit] relatively short, stout projection with a rounded tip, on the gonopod

(gonopodal) **supplementary spur**/spurs: [Lit] spur on the female gonopod other than those invariantly present

**lateral denticle**/denticles (of gonopodal claw/s): [Lit] one of the denticles on one or both sides of a gonopodal claw. Fig. 61. Syn.: (gonopodal) lateral claw/claws

**gonopodal lamina**: [Geo] entire median projection, corresponding to the paired female gonopods. Fig. 62. Syn.: genital appendage

**penis**: median projection bearing the male genital pore. Fig. 54. Syn.: aedeagus, intromittent apparatus, median lobe, spinneret

**postpedal tergite**: the most posterior tergite of the trunk. Fig. 49. Syn.: anal tergite, tergite of telson, tergum of postpedal segments

**lamina adanalis**: [Sco] median dorsal flat projection on the posterior tip of the female body. Fig. 63. Syn.: adanal lamina

**lamina subanalis**: [Sco] median ventral flat projection on the posterior tip of the female body. Fig. 63

**subanal plate**/plates: [Scu] one of the paired ventrolateral sclerites in the telson. Fig. 59

**anogenital capsule**: [Cra] terminal, capsule-like part of the trunk. Fig. 64. Syn.: anal capsule

**anal pore**/pores: [Cra, Geo, Lit] one of the pores of the anal organs, on the ventro-lateral sides of the telson. Fig. 51. Syn.: terminal pore/pores

**anal valve**/valves: one of the paired rounded flat projections on the ventral side of the telson. Fig. 58

### Abbreviations and formulae

Conventional abbreviations recommended for describing particular elements and patterns of elements are described below.

**Antennal articles in Geophilomorpha**. Each article is indicated by a Roman number, from the most basal article (I) to the most distal one (XIV).

**Arrangement of ocelli in Lithobiomorpha**. The number of ocelli in different rows are indicated from the most dorsal row to the most ventral row, separated by commas [1+ n_1_, n_2_, … where 1 is the posterior ocellus and n_1_, n_2_, …are the numbers of seriate ocelli in the rows].

**Pattern of teeth on the anterior margin of the forcipular coxosternite in Lithobiomorpha and on the tooth-plates in Scolopendromorpha**. The number of teeth is indicated for the right and the left side, separated by a plus [n_right_ + n_left_].

**Leg-bearing segments and pairs of legs**. Each leg-bearing segment, or the corresponding pair of legs, is indicated by an Arabic number, from the most anterior one (1) to the most posterior one.

**Tergites and sternites of the leg-bearing segments**. Each tergite and sternite is indicated by T and S respectively (TT and SS for multiple tergites and sternites, respectively), followed by an Arabic number, from the most anterior ones (T1 and S1) to the most posterior ones.

**Arrangement of spurs on the legs in Lithobiomorpha** (plectrotaxy; Table 3). The position of the spurs on each article of the legs is indicated in a tabular form (Table 4). Spurs on the ventral side are indicated on the left part of the table, those on the dorsal side on the right part. The pairs of legs are indicated by Arabic numbers, from the most anterior one (1) to the most posterior one (15), as described above. The articles are indicated by the following abbreviations: C = coxa, t = trochanter, P = prefemur, F = femur, T = tibia (upper case letter, except for trochanter). The position of each spur relative to the antero-posterior axis is indicated by the following abbreviations: a = anterior, m = median, p = posterior (lower case letter). Leg articles without spurs are indicated by a dash. Spurs that could be absent (variation) are indicated in parentheses. Spurs that could be present on one side only are marked by an asterisk. Additional spurs are indicated by the abbreviation of their position (a, m, p) typed as a superscript to the corresponding spur (e.g., a^a^). Legs with the same plectrotaxy in both ventral and dorsal side can be indicated in distinct rows or in a single, common line. This convention was first proposed by Ribaut (1921) and introduced in the English literature by E.H. Eason and R.E. Crabill (Eason 1951, 1964; Crabill 1953, 1962; Crabill and Lorenzo 1957). Each individual spur is indicated in a text with a formula comprising the following abbreviations: the pair of legs (1–15), the side of the leg (V = ventral, D = dorsal), the position relative to the antero-posterior axis (a, m, p), the leg article (C, t, P, F, T) (e.g., 15VaC).

**Pattern of coxal pores on the legs in Lithobiomorpha**. The number of coxal pores is indicated from anterior to posterior legs, without separation between the numbers […n_13_n_14_n_15_].

**Table 4.** Example of conventional table describing the plectrotaxy of Lithobius forficatus. See text for abbreviations.

	ventral					dorsal				
	C	t	P	F	T	C	t	P	F	T
1	-	-	mp	amp	am	-	-	mp	ap	a
2	-	-	mp	amp	am	-	-	amp	ap	ap
3	-	-	mp	amp	am	-	-	amp	ap	ap
4	-	-	mp	amp	am	-	-	amp	ap	ap
5	-	-	mp	amp	am	-	-	amp	ap	ap
6	-	-	mp	amp	am	-	-	amp	ap	ap
7	-	-	mp	amp	am	-	-	amp	ap	ap
8	-	-	mp	amp	am	-	-	amp	ap	ap
9	-	-	mp	amp	am	-	-	amp	ap	ap
10	-	-	mp	amp	am	-	-	amp	ap	ap
11	-	-	mp	amp	am	-	-	amp	ap	ap
12	-	-	amp	amp	am	a	-	amp	p	ap
13	-	m	amp	amp	am	a	-	amp	p	p
14	-	m	amp	amp	am	a	-	amp	p	p
15	-	m	amp	amp	am	a	-	amp	p	-

## Supplementary materials

Appendix I. **Pre-1981 publications.** Selected publications published before 1981 from which morphological terms have been retrieved. File format: PDF. doi: 10.3897/zookeys.69.737-app.I

Appendix II. **Analytical index.** Alphabetic index of morphological terms used for Chilopoda in the English literature. File format: PDF. doi: 10.3897/zookeys.69.737-app.II
