# Supplementary material for: A common terminology for the external anatomy of centipedes (Chilopoda)
Source: Zookeys. 2010 Nov 18;(69):17–51. doi: 10.3897/zookeys.69.737 (PMC3088443; doi:10.3897/zookeys.69.737)
Supplement: Appendix I. — Pre-1981 publications. Selected publications published before 1981 from which morphological terms have been retrieved. File format: PDF. doi: 10.3897/zookeys.69.737-app.I [file ZooKeys-069-017-SD1.pdf]

ZooKeys 69: 17–51 (2010)

# A common terminology for the external anatomy of centipedes (Chilopoda)

Lucio Bonato, Gregory D. Edgecombe, John G.E. Lewis, Alessandro Minelli, Luis A. Pereira, Rowland M. Shelley, Marzio Zapparoli

## Appendix I

## Pre-1981 publications

Selected publications, from among those that appeared before Lewis (1981), from which morphological terms have been retrieved, in addition to all later publications (see under Methods).

ARCHEY G, 1936, *Rec. Auckland Inst. Mus.*, 2: 43-70; 1937, *Rec. Auckland Inst. Mus.*, 2: 71-100. ATTEMS C, 1928, *Ann. South African Mus.*, 26: 1-431. BLOWER JG, 1951, *Quart. J. Micr. Sci.*, 92: 141-161; 1961, *Ann. Mag. Nat. Hist.*, 4: 183-187. BRADE-BIRKS SG, 1939, *J. South-Eastern Agr. Coll.*, 44: 156-179. CAPUSE I, 1975, *Trav. Inst. Spéol. E. Racovitza*, 14: 35-44. CHAMBERLIN RV, 1910, *Pomona Coll. J. Entom.*, 2: 363-374; 1911, *Ann. Entom. Soc. America*, 4: 32-48; 1912, *Bull. Mus. Compar. Zool., Harvard Coll.*, 54: 405-436; 1912, *Bull. Mus. Compar. Zool., Harvard Coll.*, 57: 1-36; 1913, *Bull. Mus. Compar. Zool., Harvard Coll.*, 57: 37-104; 1914, *Bull. Mus. Compar. Zool., Harvard Coll.*, 57: 105-112; 1914, *Bull. Mus. Compar. Zool., Harvard Coll.*, 58: 151-221; 1915, *Bull. Mus. Compar. Zool., Harvard Coll.*, 59: 493-541; 1917, *Bull. Mus. Compar. Zool., Harvard Coll.*, 57: 209-255; 1920, *Bull. Mus. Compar. Zool., Harvard Coll.*, 64: 1-269; 1922, *Bull. Mus. Compar. Zool., Harvard Coll.*, 57: 259-382; 1925, *Bull. Mus. Compar. Zool., Harvard Coll.*, 57: 441-504; 1944, *Field Mus. Nat. Hist. Publ., Zool. Ser.*, 28: 175-216; 1952, *Istanbul Üniv. Fen Fak. Mecm.*, 17B: 183-258; 1955, *Lunds Univ. Ärsskrift, N.F. Avd.* 2, 51: 1-61; 1962, *Univ. Utah Biol. Ser.*, 12: 1-23. CRABILL RE, 1952, *Entom. News*, 63: 203-206; 1954, *Proc. Entom. Soc. Washington*, 56: 172-188; 1958, *Entom. News*, 69: 153-160; 1958, *J. Washington Acad. Sci.*, 48: 260-262; 1959, *J. Washington Acad. Sci.*, 49: 188-192; 1959, *Pacific insects*, 1: 173-176; 1960, *Proc. U. S. Nat. Mus.*, 111: 1-15; 1960, *Proc. U. S. Nat. Mus.*, 111: 167-195; 1960, *Proc. Biol. Soc. Washington*, 73: 87-94; 1961, *Bull. Brooklyn Entom. Soc.*, 55: 121-133; 1961, *Senckenb. Biol.*, 42: 501-505; 1961, *Entom. News*, 72: 155-159, 177-190; 1961, *Proc. Entom. Soc. Washington*, 63: 125-135; 1962, *Proc. U. S. Nat. Mus.*, 113: 399-412; 1964, *Proc. Biol. Soc. Washington*, 77: 161-170; 1964, *Entom. News*, 75: 33-42; 1969, *Entom. News*, 80: 38-43; 1970, *J. Nat. Hist.*, 4: 231-237. CRABILL RE and LORENZO MA, 1957, *Canadian Entom.*, 89: 428-432. EASON EH, 1964, Centipedes of the British Isles, *Warne & Co*; 1970, *Bull. Mus. Nat. Hist. Nat., Paris*, 41, suppl. 2: 58-60. LAWRENCE RF, 1953, The biology of cryptic fauna of forests with special reference to the indigenous forests of South Africa, *A.A. Balkema*; 1955, *Ann. Natal Mus.*, 13: 121-174; 1958, *Ann. Natal Mus.*, 14: 279-301; 1959, *Ann. Transvaal Mus.*, 23: 363-386; 1963, *Ann. Natal Mus.*, 15: 297-318; 1975, *Cimbebasia*, 4: 35-45. LEWIS JGE, 1963, *Ann. Mag. Nat. Hist.*, 6B, 49-55; 1967, *Proc. Linn. Soc. London*, 178: 185-207. MANTON SM, 1958, *J. Linn. Soc. London, Zool.*, 43: 487-556; 1964, *Phil. Trans. R. Soc. London*, 247B, 1-183; 1965, *J. Linn. Soc. London, Zool.*, 46: 251-484; 1974, *Symp. Zool. Soc. London*, 32: 163-190. SHINOHARA K, 1961, *Zool. Mag.*, 70: 212-216. SILVESTRI F, 1917, *Rec. Indian Mus.*, 13: 307-314; 1919, *Rec. Indian Mus.*, 16: 45-107; 1929, *Rec. Indian Mus.*, 31: 263-267. TAKAKUWA Y, 1938, *Bot. Zool.*, 6: 2023-2032. TAKAKUWA Y and TAKASHIMA H, 1949, *Acta Arachn.*, 11: 51-69. TURK FA, 1955, *Proc. Zool. Soc. London*, 125: 469-504. VERHOEFF KW, 1925, *Ark. Zool.*, 17A, 1-62. WANG YM, 1951, *Serica*, 1; 1962, *Quart. J. Taiwan Mus.*, 15: 79-106. WÜRMLI M, 1974, *Symp. Zool. Soc. London*, 32: 89-98; 1979, Myriapod biology (Camatini M, ed.), *Academic Press*, 39-48. ZALESSKAJA NT, 1975, *Zool. Zh.*, 54: 1316-1325; 1976, *Zool. Zh.*, 55: 607-611.
