# Supplementary material for: A common terminology for the external anatomy of centipedes (Chilopoda)
Source: Zookeys. 2010 Nov 18;(69):17–51. doi: 10.3897/zookeys.69.737 (PMC3088443; doi:10.3897/zookeys.69.737)
Supplement: Appendix II. — Analytical index. Alphabetic index of morphological terms used for Chilopoda in the English literature. File format: PDF. doi: 10.3897/zookeys.69.737-app.II [file ZooKeys-069-017-SD2.pdf]

ZooKeys 69: 17–51 (2010)

A common terminology for the external anatomy of centipedes (Chilopoda)

Lucio Bonato, Gregory D. Edgecombe, John G.E. Lewis, Alessandro Minelli, Luis A. Pereira, Rowland M. Shelley, Marzio Zapparoli

Appendix II

Analytical index

Alphabetic index of morphological terms used for Chilopoda in the English literature. For recommended terms (in bold), the number of page or Table is given where it is defined, together with the figure where it is illustrated. Alternative terms (not in bold) point to the corresponding recommended terms.

acicula/aciculae, (mandibular): 27, fig. 15
aedeagus → **penis**: 46, fig. 54
ala/alae, (labral): 26, fig. 8
  ala/alae, (labral), **anterior**: 26, fig. 8
  ala/alae, (labral), **posterior**: 26, fig. 8
alveolus/alveoli, setal → **socket**/sockets, **setal**: Tab. 1
angulation/angulations (of tergite) → **projection**/projections (of tergite), **posterior triangular**: 36, fig. 34
annulation/annulations, (antennal): 23, fig. 11
  annulation/annulations, (tarsal): 40, fig. 45
annulus/annuli, (antennal) → **article**/articles, (antennal): 23, fig. 1
  annulus/annuli, (tarsal) → **annulation**/annulations, (tarsal): 40, fig. 45
antenna/antennae: 23, fig. 1
antennomere/antennomeres → **article**/articles, (antennal): 23, fig. 1
apparatus, intromittent → **penis**: 46, fig. 54
appendage/appendages, genital → **gonopod**/gonopods: 45, fig. 58
  appendage, genital → **lamina**, **gonopodal**: 46, fig. 62
arc, (labral) **median**: 26, fig. 12
arch, labral → **arc**, (labral) **median**: 26, fig. 12
area/areas, anteroclypeal → **area**/areas, **clypeal**: 25, fig. 7
  area/areas, **clypeal**: 25, fig. 7
  area/areas, (clypeal or prelabral) consolidated → **plagula**/plagulae: 25, fig. 9
  area/areas, (coxal) cribriform → **pore-field**/pore-fields, (coxal): 43, fig. 52
  area/areas, (coxal) porose → **pore-field**/pore-fields, (coxal): 43, fig. 52
  area/areas, eye → **area**/areas, **ocellar**: 21, fig. 3
  area/areas, **ocellar**: 21, fig. 3
  area/areas, ocellary → **area**/areas, **ocellar**: 21, fig. 3
  area/areas, ocular → **area**/areas, **ocellar**: 21, fig. 3
  area/areas, pore → **pore-field**/pore-fields, (ventral): 39, fig. 40
  area/areas, poriferous → **pore-field**/pore-fields, (ventral): 39, fig. 40
  area/areas, porigerous → **pore-field**/pore-fields, (ventral): 39, fig. 40
areolation: Tab. 3
armature → **plectrotaxy**: Tab. 3
article/articles, (antennal): 23, fig. 1
  article/articles, (antennal) → **annulation**/annulations, (antennal): 23, fig. 11
  article/articles, (first maxillary) **basal**: 29, fig. 18
  article/articles, (first maxillary), **distal**: 29, fig. 18
  article/articles, first tarsal → **tarsus**/tarsi **1**: 40, fig. 44
  article/articles, (forcipular) **intermediate**: 35
  article/articles, (forcipular or prehensorial) basal → **trochanteroprefemur**/trochanteroprefemora, (forcipular): 35, figs 29, 32
  article/articles, (forcipular or prehensorial) first → **trochanteroprefemur**/trochanteroprefemora, (forcipular): 35, figs 29, 32

article/articles, (forcipular or prehensorial) intercalary → **article**/articles, (forcipular) **intermediate**: 35
article/articles, (forcipular or prehensorial) second → **femur**/femora, (forcipular): 35, figs 29, 32
article/articles, (forcipular or prehensorial) third → **tibia**/tibiae, (forcipular): 35, figs 29, 32
article/articles, (gonopodal) basal → **article**/articles, (gonopodal) **first**: 45, fig. 61
article/articles, (gonopodal) **first**: 45, fig. 61
article/articles, (gonopodal) **second**: 46, fig. 61
article/articles, (gonopodal) **third**: 46, fig. 61
article/articles, (leg): 40, fig. 41
article/articles (second maxillary), apical → **article**/articles **3**, (second maxillary): 32, fig. 23
article/articles (second maxillary), basal → **article**/articles **1**, (second maxillary): 32, fig. 23
article/articles (second maxillary), first → **article**/articles **1**, (second maxillary): 32, fig. 23
article/articles (second maxillary), second → **article**/articles **2**, (second maxillary): 32, fig. 23
article/articles (second maxillary), third → **article**/articles **3**, (second maxillary): 32, fig. 23
article/articles (second maxillary), ultimate → **article**/articles **3**, (second maxillary): 32, fig. 23
article/articles, second tarsal → **tarsus**/tarsi **2**: 40, fig. 44
article/articles, secondary (tarsal) → **annulation**/annulations, (tarsal): 40, fig. 45
article/articles, **tarsal**: 40
article/articles 1, (forcipular or prehensorial) → **trochanteroprefemur**/trochanteroprefemora, (forcipular): 35, figs 29, 32
article/articles **1**, (second maxillary): 32, fig. 23
article/articles 2, (forcipular or prehensorial) → **femur**/femora, (forcipular): 35, figs 29, 32
article/articles **2**, (second maxillary): 32, fig. 23
article/articles 3, (forcipular or prehensorial) → **tibia**/tibiae, (forcipular): 35, figs 29, 32
article/articles **3**, (second maxillary): 32, fig. 23
article/articles 4, (forcipular or prehensorial) → **tarsungulum**/tarsungula, (forcipular): 35, fig. 32
basitarsus/basitarsi → **tarsus**/tarsi **1**: 40, fig. 44
block/blocks, (mandibular): 27, fig. 17
body/bodies, (mandibular) → **trunk**/trunks, (mandibular): 27, fig. 15
border/borders (of tergite) → **margination**/marginations (of tergite): 36, fig. 34
branch/branches, (first maxillary) inner → **projection**/projections, (first maxillary) **coxal**: 29, fig. 21
branch/branches, (first maxillary) outer → **telopodite**/telopodites, (first maxillary): 29, fig. 21
branch/branches **of antennocellar suture**, **antennal**: 21
branch/branches **of antennocellar suture**, **ocellar**: 21
bristle/bristles → **seta**/setae: Tab. 2
bristle/bristles, basal → **spine**/spines, **accessory**: 41, fig. 47
bristle/bristles, (labral): 26, fig. 14
bristle/bristles, (labral) branching → **bristle**/bristles, (labral): 26, fig. 14
bristle/bristles, (mandibular) **branching**: 27
bristle/bristles, (first maxillary) **plumose**: 29, fig. 21
bristle/bristles, sickle → **acicula**/aciculae, (mandibular): 27, fig. 15
bristle/bristles, sickle-shaped → **acicula**/aciculae, (mandibular): 27, fig. 15
brush/brushes, (second maxillary) **dorsal**: 32, fig. 22
bucca/buccae → **pleurite**/pleurites, **cephalic**: 23, fig. 7
bulge, (cephalic) marginal → **ridge**, (cephalic) **marginal**: 23, fig. 1
calcar/calcars → **spur**/spurs, (leg): 40, fig. 44
capsule, anal → **capsule**, **anogenital**: 46, fig. 64
capsule, **anogenital**: 46, fig. 64
capsule, **cephalic**: 20, fig.1
capsule, head → **capsule**, **cephalic**: 20, fig. 1
carina/carinae: 40, fig. 46
carpophagus-structure/carpophagus-structures: 39, fig. 37
catapleurite/catapleurites: 37, fig. 36
catopleure/catopleures → **catapleurite**/catapleurites: 37, fig. 36
cavity/cavities, (coxopleural or porigerous) → **fossa**/fossae, (coxopleural): 43
cerrus/cerri, (coxosternal): 33, fig. 27
chaetotaxy → **setation**: Tab. 3
chitin-line/chitin-lines: 33, fig. 30
claw/claws: 40, fig. 44
claw/claws, accessory → **spine**/spines, **accessory**: 41, fig. 47
claw/claws, anterior accessory → **spine**/spines, **anterior accessory**: 41, fig. 47

claw/claws, apical → **claw**/claws: 40, fig. 44
claw/claws, end → **claw**/claws: 40, fig. 44
claw/claws, (forcipular or prehensorial) → **tarsungulum**/tarsungula, (forcipular): 35, fig. 32
claw/claws, (gonopodal): 46, fig. 61
claw/claws, (gonopodal) lateral → **denticle**/denticles (of gonopodal claw/s), **lateral**: 46, fig. 61
claw/claws, main → **unguis**/ungues **proper**: 41, fig. 47
claw/claws, poison → **forcipule**/forcipules: 35, fig. 28
claw/claws, poison → **tarsungulum**/tarsungula, (forcipular): 35, fig. 32
claw/claws, posterior accessory → **spine**/spines, **posterior accessory**: 41, fig. 47
claw/claws, principal → **unguis**/ungues **proper**: 41, fig. 47
claw/claws proper → **unguis**/ungues **proper**: 41, fig. 47
claw/claws, (second maxillary): 32, fig. 26
claw/claws, (second maxillary) apical → **claw**/claws, (second maxillary): 32, fig. 26
claw/claws, (second maxillary) pretarsal → **claw**/claws, (second maxillary): 32, fig. 26
claw/claws, (second maxillary) terminal → **claw**/claws, (second maxillary): 32, fig. 26
claw/claws, tarsal → **claw**/claws: 40, fig. 44
cleft, **median** (coxosternal): 33, fig. 28
cleft/clefts, parasternital → **gutter**/gutters (of sternite), **lateral**: 39, fig. 42
clypeolabrum: 25, fig. 7
clypeus: 25, fig. 12
clypeus, areolate → **part** (of clypeus), **areolate**: 25, fig. 9
clypeus, posterior → **plagula**/plagulae: 25, fig. 9
coclypeus/coclypei → **sclerite**/sclerites, **paralabial**: 26, fig. 13
collar, (forcipular): 33, fig. 28
collar, (maxillipede) (pleural) → **collar**, (forcipular): 33, fig. 28
comb/combs, (forcipular) **spine**: 35
comb/combs (of second maxillary claw): 32, fig. 18
comb/combs of teeth → **comb**/combs (of second maxillary claw): 32, fig. 18
comb/combs, (tibial and tarsal) (serrate) → **tooth**/teeth**, saw**: 43, fig. 56
complex, **maxillary**: 23
complex, tentorial → **tentorium**/tentoria: 26, fig. 13
condyle/condyles, (coxofemoral or prehensorial) → **condyle**/condyles, **coxosternal**: 33, fig. 30
condyle/condyles, **coxosternal**: 33, fig. 30
condyle/condyles, **mandibular**: 26, fig. 15
corpus/corpora, (mandibular) → **trunk**/trunks, (mandibular): 27, fig. 15
coxa/coxae, (anal or last) → **coxopleuron**/coxopleura: 43, fig. 52
coxa/coxae, (first maxillary): 29
coxa/coxae, (forcipular): 33, fig. 29
coxa/coxae, (gonopodal) → **article**/articles, (gonopodal) **first**: 45, fig. 61
coxa/coxae, (leg): 40, fig. 38
coxae, (first maxillary) → **coxosternite**, (first maxillary): 29, fig. 19
coxite/coxites, **first genital**: 45, fig. 59
coxite/coxites, (forcipular) → **coxa**/coxae, (forcipular): 33, fig. 29
coxite/coxites, (gonopodal) → **article**/articles, (gonopodal) **first**: 45, fig. 61
coxites, (first maxillary) → **coxosternite**, (first maxillary): 29, fig. 19
coxopleura/coxopleurae → **coxopleuron**/coxopleura: 43, fig. 52
coxopleurite/coxopleurites → **coxopleuron**/coxopleura: 43, fig. 52
coxopleuron/coxopleura: 43, fig. 52
coxosterna, (first maxillary) → **coxosternite**, (first maxillary): 29, fig. 19
coxosterna, (second maxillary) → **coxosternite**, (second maxillary): 31, fig. 23
coxosternite, (first maxillary): 29, fig. 19
coxosternite, (forcipular): 33, fig. 28
coxosternite, (maxillipede) → **coxosternite**, (forcipular): 33, fig. 28
coxosternite, (second maxillary): 31, fig. 23
coxosternum, (first maxillary) → **coxosternite**, (first maxillary): 29, fig. 19
coxosternum, (forcipular or maxillipede) → **coxosternite**, (forcipular): 33, fig. 28
coxosternum, (second maxillary) → **coxosternite**, (second maxillary): 31, fig. 23
crypt/crypts, (coxal or coxopleural) → **pit**/pits, (coxopleural): 43, fig. 54
cursiped/cursipeds: 39

denticle/denticles, (coxosternal or prosternal) (anterior or anterocentral) → **tooth**/teeth, (coxosternal): 33, fig. 32
denticle/denticles, (coxosternal): 33, fig. 30
denticle/denticles, (forcipular): 35, figs 27, 30
denticle/denticles, (gonopodal) (accessory) → **spur**/spurs, (gonopodal): 46, fig. 61
denticle/denticles, (labral): 26, fig. 12
denticle/denticles, (mandibular) **accessory**: 27
denticle/denticles (of gonopodal claw/s), **lateral**: 46, fig. 61
denticle/denticles (of tarsungulum), **basal**: 35, fig. 30
denticle/denticles (of trochanteroprefemur), basal → **denticle**/denticles (of trochanteroprefemur), **proximal**: 35, fig. 27
denticle/denticles (of trochanteroprefemur), **distal**: 35, fig. 27
denticle/denticles (of trochanteroprefemur), **proximal**: 35, fig. 27
dentition, (coxosternal): Tab. 3
depression/depressions: Tab. 1
depression/depressions, (coxal) **gutter-like**: 43, fig. 53
depression/depressions, paired posterior → **sulcus**/sulci, (cephalic) **paramedian**: 23, fig. 4
depression/depressions **of proarthron**: 45, fig. 59
diastema, (coxosternal) **median**: 33, fig. 31
digit/digits (of second maxillary claw): 32, fig. 26
disjuncture/disjunctures of limbus → **interruption**/interruptions (of cephalic plate), **lateral marginal**: 23
distitarsus/distitarsi → **tarsus**/tarsi **2**: 40, fig. 44
distotarsus/distotarsi → **tarsus**/tarsi **2**: 40, fig. 44
division/divisions of antenna/antennae, first → **flagellum**/flagella, **first**: 25, fig. 10
division/divisions of antenna/antennae, second → **flagellum**/flagella, **second**: 25, fig. 11
division/divisions of antenna/antennae, third → **flagellum**/flagella, **third**: 25, fig. 11
division/divisions of tarsus/tarsi, first → **tarsus**/tarsi **1**: 40, fig. 44
division/divisions of tarsus/tarsi, second → **tarsus**/tarsi **2**: 40, fig. 44
duploflagellum/duploflagella → **flagellum**/flagella: 25, figs 10,11
ectodont/ectodonts → **porodont**/porodonts: 33, fig. 31
edge/edges, **gnathal**: 26, fig. 16
edge/edges, molar → **edge**/edges, **gnathal**: 26, fig. 16
embayment, **median** (coxosternal): 35, fig. 28
endite/endites, (forcipular or maxillipede) coxal → **shoulder**/shoulders (of forcipular coxosternite): 33, fig. 31
endosternite/endosternites: 39, fig. 37
eucoxa/eucoxae **inferior**/inferiores: 37
eucoxa/eucoxae **superior**/superiores: 37
eupleurium/eupleuria: 36
eye/eyes, **compound**: 21, fig. 3
femoroid/femoroids, (first maxillary) → **article**/articles, (first maxillary) **basal**: 29, fig. 18
femoroid/femoroids, (forcipular or prehensorial) → **femur**/femora, (forcipular): 35, figs 29, 32
femoroid/femoroids, (forcipular or prehensorial) → **trochanteroprefemur**/trochanteroprefemora, (forcipular): 35, figs 29, 32
femoroid/femoroids, (second maxillary) → **article**/articles **1**, (second maxillary): 32, fig. 23
femur/femora, (forcipular): 35, figs 29, 32
femur/femora, (leg): 40, fig. 43
femur/femora, (second maxillary): 31, fig. 25
femuroid/femuroids, (forcipular or prehensorial) → **femur**/femora, (forcipular): 35, figs 29, 32
femuroid/femuroids, (forcipular or prehensorial) → **trochanteroprefemur**/trochanteroprefemora, (forcipular): 35, figs 29, 32
fenestra/fenestrae, (anterocentral or clypeal) → **area**/areas, **clypeal**: 25, fig. 7
fibulunguis/fibulungues: 41, fig. 48
field/fields, (clypeal or prelabral) non-areolate → **plagula**/plagulae: 25, fig. 9
field/fields, ocellary → **area**/areas, **ocellar**: 21, fig. 3
filament/filaments, (labral) → **bristle**/bristles, (labral): 26, fig. 14
filament/filaments (of second maxillary claw): 32, fig. 18
fimbria/fimbriae, (labral) (branched) → **bristle**/bristles, (labral): 26, fig. 14
fissure/fissures, cruciform → **suture**/sutures, (mandibular) **cruciform**: 27, fig. 16
flagellum/flagella: 25, figs 10,11
flagellum/flagella, **first**: 25, fig. 10
flagellum/flagella primum/prima → **flagellum**/flagella, **first**: 25, fig. 10
flagellum/flagella secundum/secunda → **flagellum**/flagella, **second**: 25, fig. 11

flagellum/flagella, **second**: 25, fig. 11
flagellum/flagella tertium/tertia → **flagellum**/flagella, **third**: 25, fig. 11
flagellum/flagella, **third**: 25, fig. 11
foot/feet, prehensorial → **forcipule**/forcipules: 35, fig. 28
forcipula/forcipulae → **forcipule**/forcipules: 35, fig. 28
forcipule/forcipules: 35, fig. 28
fossa/fossae: Tab. 1
fossa/fossae, carpophagus → **pit**/pits, **carpophagus**: 39, fig. 37
fossa/fossae, (coxopleural): 43
fossa/fossae (of sternite), **transverse**: 39, fig. 41
fossa/fossae, parasternital → **gutter**/gutters (of sternite), **lateral**: 39, fig. 42
fossa/fossae, **virguliform**: 39, fig. 42
fossula/fossulae, (coxopleural or porigerous) → **fossa**/fossae, (coxopleural): 43
fovea/foveae, **fungiform**: 39
frons → **plate**, **frontal**: 21, fig. 4
fulcrum/fulcra, (labral or mandibular) → **tentorium**/tentoria: 26, fig. 13
fultura/fulturae, (labral or labial) → **tentorium**/tentoria: 26, fig. 13
fundus/fundi (of claw): 41, fig. 47
furrow/furrows → **sulcus**/sulci: Tab. 1
furrow, (cephalic median) → **sulcus**, (cephalic) **median**: 20, fig. 1
furrow/furrows (of sternite), cross → **suture**/sutures (of sternite), **cruciform**: 39, fig. 39
gland-pit/gland-pits, subsurface → **pit**/pits, (coxopleural): 43, fig. 54
gonopod/gonopods: 45, fig. 58
groove/grooves (of tergite), paramedian → **sulcus**/sulci or **suture**/sutures (of tergite), **paramedian**: 36, fig. 33
groove/grooves (of tergite), (procurved) cervical → **sulcus**/sulci **or suture**/sutures (of tergite), (anterior) **transverse**: 36, fig. 33
groove/grooves (of tergite), (procurved) transverse → **sulcus**/sulci **or suture**/sutures (of tergite), (anterior) **transverse**: 36, fig. 33
gutter/gutters → **depression**/depressions: Tab. 1
gutter/gutters (of sternite), **lateral**: 39, fig. 42
Haarpolster → **pulvillus**/pulvilli: 27, fig. 15
hair/hairs: Tab. 2
hair/hairs → **seta**/setae: Tab. 2
hair/hairs, (labral) → **bristle**/bristles, (labral): 26, fig. 14
impression/impressions (of sternite), cruciform → **suture**/sutures (of sternite), **cruciform**: 39, fig. 39
incision/incisions, (first maxillary) **lateral**: 29, fig. 20
incision/incisions (of stilus), **anterior**: 23, fig. 8
insula/insulae, **clypeal**: 25, fig. 9
insula/insulae, (second maxillary) **setigerous**: 31, fig. 20
interruption/interruptions (of cephalic plate), **lateral marginal**: 23
intersternite/intersternites → **presternite**/presternites: 37, fig. 37
intertergite/intertergites → **pretergite**/pretergites: 36, fig. 33
interval, (coxosternal) median → **diastema**, **median**: 33, fig. 31
isthmus, (second maxillary): 31, fig. 24
joint/joints, (antennal) → **article**/articles, (antennal): 23, fig. 1
joint/joints, first tarsal → **tarsus**/tarsi **1**: 40, fig. 44
joint/joints, (forcipular or prehensorial) second → **femur**/femora, (forcipular): 35, figs 29, 32
joint/joints, (forcipular or prehensorial) third → **tibia**/tibiae, (forcipular): 35, figs 29, 32
joint/joints, (forcipular or prehensorial) intermediate → **article**/articles, (forcipular) **intermediate**: 35, fig. 29
joint/joints (second maxillary), second → **article**/articles **2**, (second maxillary): 32, fig. 23
joint/joints (second maxillary), terminal → **article**/articles **3**, (second maxillary): 32, fig. 23
joint/joints, second tarsal → **tarsus**/tarsi **2**: 40, fig. 44
katopleure/katopleures → **catapleurite**/catapleurites: 37, fig. 36
labium → **maxillae**, **second**: 31, fig. 18
labromere, median → (labral) **intermediate part**: 26, fig. 14
labrum: 25, fig. 12
lamella/lamellae dentata/dentatae → **lamella**/lamellae, (mandibular) **dentate**: 27, fig. 17
lamella/lamellae, (mandibular): 27, fig. 17
lamella/lamellae, (mandibular) **dentate**: 27, fig. 17

lamella/lamellae, (mandibular) **pectinate**: 27, fig. 17
lamina, adanal → **lamina adanalis**: 46, fig. 63
lamina adanalis: 46, fig. 63
lamina basalis → **pretergite**, **forcipular**: 32, fig. 2
lamina/laminae **condylifera**/condyliferae: 27, fig. 16
lamina/laminae **dentifera**/dentiferae: 27, fig. 16
lamina, **gonopodal**: 46, fig. 62
lamina/laminae, (mandibular) → **lamella**/lamellae, (mandibular): 27, fig. 17
lamina/laminae, (mandibular) dentate → **lamella**/lamellae, **dentate**: 27, fig. 17
lamina/laminae **manubrii**: 27, fig. 16
lamina/laminae, pectinate → **lamella**/lamellae, **pectinate**: 27, fig. 17
lamina subanalis: 46, fig. 63
lamina/laminae **triangularis**/triangulares: 27, fig. 16
lappet/lappets, (first maxillary): 29, fig. 19
lappet/lappets, (first maxillary) **coxosternal**: 29, fig. 19
lappet/lappets, (first maxillary) external sensory → **lappet**/lappets, (first maxillary): 29, fig. 19
lappet/lappets, (first maxillary) syncoxital → **lappet**/lappets, (first maxillary) **coxosternal**: 29, fig. 19
lappet/lappets, (first maxillary) **telopodital**: 29, fig. 19
leg/legs: 39, fig. 41
leg/legs, (ambulatory or locomotory or walking) → **leg**/legs: 39, fig. 41
leg/legs, anal → **leg**/legs, **ultimate**: 43, fig. 49
leg/legs, caudal → **leg**/legs, **ultimate**: 43, fig. 49
leg/legs, end → **leg**/legs, **ultimate**: 43, fig. 49
leg/legs, last → **leg**/legs, **ultimate**: 43, fig. 49
leg/legs, penult → **leg**/legs, **penultimate**: 41, fig. 49
leg/legs, **penultimate**: 41, fig. 49
leg/legs, posterior → **leg**/legs, **ultimate**: 43, fig. 49
leg/legs, terminal → **leg**/legs, **ultimate**: 43, fig. 49
leg/legs, **ultimate**: 43, fig. 49
limb/limbs of (cephalic) transverse suture, posterior → **branch**/branches **of antennocellar suture**, **ocellar**: 21
limbus → **ridge**, (cephalic) **marginal**: 23, fig. 1
line/lines, chitinous → **chitin-line**/chitin-lines: 33, fig. 30
line, frontal → **suture**, (cephalic) **transverse**: 21, figs 3,4
line/lines, hinge → **suture**/sutures: Tab. 1
line/lines, (labral) **transverse thickened**: 26, fig. 8
line/lines, (second mexillary) pleurocoxal → **statumen**/statuminia: 31, fig. 24
lip, upper → **labrum**: 25, fig. 12
lobe/lobes, (first maxillary) inner → **projection**/projections, (first maxillary) **coxal**: 29, fig. 21
lobe/lobes, (first maxillary) medial → **projection**/projections, (first maxillary) **coxal**: 29, fig. 21
lobe/lobes, (first maxillary) outer → **telopodite**/telopodites, (first maxillary): 29, fig. 21
lobe/lobes, (first maxillary) syncoxal → **lappet**/lappets, (first maxillary) **coxosternal**: 29, fig. 19
lobe/lobes, gnathal → **edge**/edges, **gnathal**: 26, fig. 16
lobe, median → **penis**: 46, fig. 54
macropore/macropores: 43, fig. 51
macroseta/macrosetae → **spine-bristle**/spine-bristles: Tab. 2
macroseta/macrosetae → **spur**/spurs, (gonopodal): 46, fig. 61
mandible/mandibles: 26, fig. 13
manubrium/manubria: 26, fig. 16
margin/margins, buccal → **stilus**/stili: 23, fig. 8
margination/marginations (of tergite): 36, fig. 34
maxilla, first → **maxillae**, **first**: 29, fig. 18
maxilla, second → **maxillae**, **second**: 31, fig. 18
maxillae, **first**: 29, fig. 18
maxillae I → **first maxillae**: 29, fig. 18
maxillae II → **second maxillae**: 31, fig. 18
maxillae, **second**: 31, fig. 18
maxilliped/maxillipeds → **forcipule**/forcipules: 35, fig. 28
maxillipede/maxillipedes → **forcipule**/forcipules: 35, fig. 28
membrane/membranes, **pleural**: 37

mesarthron: 45, fig. 59
metacoxa/metacoxae: 37, fig. 37
metarthron: 45, fig. 59
metasternite/metasternites: 39, fig. 37
metasternite/metasternites → **endosternite**/endosternites: 39, fig. 37
metasternite of the last leg-bearing segment → **metasternite of the ultimate leg-bearing segment**: 41, fig. 51
metasternite of the last trunk segment → **metasternite of the ultimate leg-bearing segment**: 41, fig. 51
metasternite of the ultimate leg-bearing segment: 41, fig. 51
metasubcoxa/metasubcoxae → **metacoxa**/metacoxae: 37, fig. 37
metatarsus/metatarsi → **tarsus**/tarsi **2**: 40, fig. 44
metatarsus/metatarsi, (forcipular or prehensorial) → **tarsungulum**/tarsungula, (forcipular): 35, fig. 32
metatergite/metatergites: 36, fig. 33
metatergite of the ultimate leg-bearing segment: 41, fig. 49
mid-piece, (labral): 25, fig. 13
mid-piece, (labral) → **part**, (labral) **intermediate**: 26, fig. 14
mucro/mucrones → **tooth**/teeth**, saw**: 43, fig. 56
node/nodes, (antennal): 25, fig. 11
node/nodes, (forcipular or prehensorial) → **denticle**/denticles, (forcipular): 35, figs 27, 30
node/nodes (of tarsungulum), (basal) → **denticle**/denticles (of tarsungulum), **basal**: 35, fig. 30
node/nodes, **porodont**: 33
nodule/nodules, (forcipular or prehensorial) → **denticle**/denticles, (forcipular): 35, figs 27, 30
notch, (coxosternal) median → **diastema**, **median**: 33, fig. 31
ocellus/ocelli: 21, fig. 5
ocellus/ocelli, major → **ocellus**/ocelli, **posterior**: 21, fig. 5
ocellus/ocelli, minor → **ocellus**/ocelli, **seriate**: 21, fig. 5
ocellus/ocelli, **posterior**: 21, fig. 5
ocellus/ocelli, **posterosuperior**: 21, fig.5
ocellus/ocelli, principal → **ocellus**/ocelli, **posterior**: 21, fig. 5
ocellus/ocelli, **seriate**: 21, fig. 5
ocellus/ocelli, terminal → **ocellus**/ocelli, **posterior**: 21, fig. 5
organ/organs of Tömösváry → **organ**/organs, **Tömösváry's**: 23, fig. 5
organ/organs, postantennal → **organ**/organs, **Tömösváry's**: 23, fig. 5
organ/organs, **shaft**: 25
organ/organs, **Tömösváry's**: 23, fig. 5
organ/organs, Tömösváry → **organ**/organs, **Tömösváry's**: 23, fig. 5
pad/pads, furry → **pulvillus**/pulvilli: 27, fig. 15
pad/pads, (first maxillary): 29, fig. 22
palisade/palisades of capitate hairs → **brush**/brushes, (second maxillary) **dorsal**: 32, fig. 22
palp/palps, (first maxillary) → **telopodite**/telopodites, (first maxillary): 29, fig. 21
palp/palps, (lateral or maxillary) → **lappet**/lappets, (first maxillary): 29, fig. 19
palp/palps, (second maxillary) → **telopodite**/telopodites, (second maxillary): 31, fig. 21
palpus/palpi, (first maxillary) → **telopodite**/telopodites, (first maxillary): 29, fig. 21
palpus/palpi, (first maxillary) coxal → **lappet**/lappets, (first maxillary) **coxosternal**: 29, fig. 19
palpus/palpi, (first maxillary) femural → **lappet**/lappets, (first maxillary) **telopodital**: 29, fig. 19
palpus/palpi, (lateral or maxillary) → **lappet**/lappets, (first maxillary): 29, fig. 19
palpus/palpi, (second maxillary) → **telopodite**/telopodites, (second maxillary): 31, fig. 21
papilla/papillae, (gonopodal): 46
papilla/papillae, **tarsal**: 40, fig. 45
parapretergite/parapretergites → **paratergite**/paratergites, **intercalary**: 37, fig. 36
paratergite/paratergites: 37, fig. 36
paratergite/paratergites, **intercalary**: 37, fig. 36
paratergite/paratergites, major → **paratergite**/paratergites, **principal**: 37, fig. 36
paratergite/paratergites, primary → **paratergite**/paratergites, **principal**: 37, fig. 36
paratergite/paratergites, **primary intercalary**: 37, fig. 36
paratergite/paratergites, **principal**: 37, fig. 36
paratergite/paratergites, secondary → **paratergite**/paratergites, **intercalary**: 37, fig. 36
paratergite/paratergites, **secondary intercalary**: 37, fig. 36
part, (clypeal) **areolate**: 25, fig. 9
part, (clypeal or prelabral) non-areolate → **plagula**/plagulae: 25, fig. 9

part, (labral) **intermediate**: 26, fig. 14
part/parts, (labral) **lateral**: 26, fig. 14
parunguis/parungues → **spine**/spines, **accessory**: 41, fig. 47
parunguis/parungues, anterior → **spine**/spines, **anterior accessory**: 41, fig. 47
parunguis/parungues, posterior → **spine**/spines, **posterior accessory**: 41, fig. 47
patellotibia/patellotibiae, (leg) → **tibia**/tibiae, (leg): 40, fig. 43
paxillus/paxilli → **peg**/pegs, **carpophagus**: 39, fig. 37
pecten/pectines, (tarsal): 40, fig. 44
peg/pegs, **carpophagus**: 39, fig. 37
penis: 46, fig. 54
piece/pieces, (labral) lateral → **side-piece**/side-pieces, (labral): 26, fig. 13
piece, (labral) median → **mid-piece**, (labral): 25, fig. 13
piece, (labral) median → **part**, (labral) **intermediate**: 26, fig. 14
piece, (labral) middle → **mid-piece**, (labral): 25, fig. 13
piece, (labral) middle → **part**, (labral) **intermediate**: 26, fig. 14
pinnule/pinnules (of acicula): 27
pit/pits → **fossa**/fossae: Tab. 1
pit/pits, **carpophagus**: 39, fig. 37
pit/pits, (coxopleural): 43, fig. 54
pit/pits, gland → **pit**/pits, (coxopleural): 43, fig. 54
pit/pits, parasternital → **gutter**/gutters (of sternite), **lateral**: 39, fig. 42
pit/pits, sternal → **pit**/pits, **carpophagus**: 39, fig. 37
plagula/plagulae: 25, fig. 9
plate/plates, anterior subcoxal → **procoxa**/procoxae: 37, fig. 37
plate, basal → **tergite**, **forcipular**: 32, fig. 2
plate, **cephalic**: 20, fig. 2
plate/plates, (cephalic) **basal**: 23, fig. 6
plate/plates, (coxosternal or prosternal) → **tooth-plate**/tooth-plates, (coxosternal): 33, fig. 32
plate/plates, (coxosternal or prosternal) dental → **tooth-plate**/tooth-plates, (coxosternal): 33, fig. 32
plate/plates, dorsal → **tergite**/tergites (of leg-bearing segment): 36, fig. 34
plate, **frontal**: 21, fig. 4
plate, head → **plate**, **cephalic**: 20, fig. 2
plate, last dorsal → **tergite** or **metatergite of the ultimate leg-bearing segment**: 41, fig. 49
plate, last ventral → **sternite** or **metasternite of the ultimate leg-bearing segment**: 41, fig. 51
plate/plates, (mandibular) dentate → **lamella**/lamellae, (mandibular) **dentate**: 27, fig. 17
plate/plates, **molar**: 27
plate, prebasal → **pretergite**, **forcipular**: 32, fig. 2
plate/plates, spiraculiferous → **stigmatopleurite**/stigmatopleurites: 37, fig. 36
plate, **subanal**: 46, fig. 59
plate/plates, tergal → **tergite**/tergites (of leg-bearing segment): 36, fig. 34
plate/plates, ventral → **sternite**/sternites (of leg-bearing segment): 37, fig. 38
plectrotaxy: Tab. 3
pleura/pleurae, cephalic → **pleurite**/pleurites, **cephalic**: 23, fig. 7
pleura/pleurae, (forcipular or maxillipede) → **pleurite**/pleurites, **forcipular**: 32, fig. 2
pleura/pleurae, (last) → **coxopleuron**/coxopleura: 43, fig. 52
pleurite/pleurites, **cephalic**: 23, fig. 7
pleurite/pleurites, **first genital**: 45, fig. 54
pleurite/pleurites, **forcipular**: 32, fig. 2
pleurite/pleurites, **intercalary**: 41, fig. 50
pleurite/pleurites, intercalary → **scutellum**/scutella: 37, fig. 36
pleurite/pleurites, **intermediate**: 45, fig. 54
pleurite/pleurites, metacoxal → **metacoxa**/metacoxae: 37, fig. 37
pleurite/pleurites of first genital segment → **pleurite**/pleurites, **first genital**: 45, fig. 54
pleurite/pleurites, procoxal → **procoxa**/procoxae: 37, fig. 37
pleurite/pleurites, spiracle-bearing → **stigmatopleurite**/stigmatopleurites: 37 fig. 36
pleurite/pleurites, stigma-bearing → **stigmatopleurite**/stigmatopleurites: 37, fig. 36
pleurite/pleurites, subcoxal → **subcoxa**/subcoxae: 37
pleurocoxa/pleurocoxae: 37
pleurocoxa/pleurocoxae → **coxopleuron**/coxopleura: 43, fig. 52

pleurogram/pleurograms → **chitin-line**/chitin-lines: 33, fig. 30
pleuron/pleura → **eupleurium**/eupleuria: 36
pleuron/pleura, cephalic → **pleurite**/pleurites, **cephalic**: 23, fig. 7
pleuron/pleura, (forcipular or maxillipede) → **pleurite**/pleurites, **forcipular**: 32, fig. 2
pleuropretergite, (ultimate): 41, fig. 49
pleurosternite, **first genital**: 45, fig. 58
plinth/plinths, (gonopodal): 46, fig. 61
pocket/pockets, subsurface → **pit**/pits, (coxopleural): 43, fig. 54
podomere/podomeres → **article**/articles, (leg): 40, fig. 41
point/points, (forcipular) **scapular**: 32, fig. 27
polygon/polygons, cuticular → **scute**/scutes: 25, fig. 7
pore/pores, **anal**: 46, fig. 51
pore/pores, **coxal**: 43, fig. 51
pore/pores, coxopleural → **pore**/pores, **coxal**: 43, fig. 51
pore/pores, **metameric**: 31, fig. 24
pore/pores, pleural → **pore**/pores, **coxal**: 43, fig. 51
pore/pores, salivary → **pore**/pores, **metameric**: 31, fig. 24
pore/pores, sternal → **pore**/pores, **ventral**: 39, fig. 40
pore/pores, sternital → **pore**/pores, **ventral**: 39, fig. 40
pore/pores, terminal → **pore**/pores, **anal**: 46, fig. 51
pore/pores, **ventral**: 39, fig. 40
pore-field/pore-fields, (coxal): 43, fig. 52
pore-field/pore-fields, (ventral): 39, fig. 40
pore-group/pore-groups → **pore-field**/pore-fields, (ventral): 39, fig. 40
porodont/porodonts: 33, fig. 31
portion/portions, (labral) lateral → **part**/parts, (labral) **lateral**: 26, fig. 14
portion/portions, (labral) lateral → **side-piece**/side-pieces, (labral): 26, fig. 13
portion, (labral) middle → **part**, (labral) **intermediate**: 26, fig. 14
portion/portions of antennocellar suture, anterior → **branch**/branches **of antennocellar suture**, **antennal**: 21
portion/portions of antennocellar suture, posterior → **branch**/branches **of antennocellar suture**, **ocellar**: 21
postarsus/postarsi → **pretarsus**/pretarsi: 40, fig. 43
posttarsus/posttarsi → **pretarsus**/pretarsi: 40, fig. 43
pouch/pouches, sternal → **fossa**/fossae, **virguliform**: 39, fig. 42
praefemur/praefemora, (leg) → **prefemur**/prefemora, (leg): 40, fig. 38
praetarsus/praetarsi → **pretarsus**/pretarsi: 40, fig. 43
praetarsus/praetarsi, (second maxillary) → **pretarsus**/pretarsi, (second maxillary): 32, fig. 23
precoxa/precoxae → **procoxa**/procoxae: 37, fig. 37
prefemur/prefemora, (leg): 40, fig. 38
prefemur/prefemora, (second maxillary): 31, fig. 25
prehensor/prehensors → **forcipule**/forcipules: 35, fig. 28
preparatergite/preparatergites → **paratergite**/paratergites, **intercalary**: 37, fig. 36
prepleurite/prepleurites → **pleurite**/pleurites, **intercalary**: 41, fig. 50
prescutellum/prescutella → **scutellum**/scutella: 37, fig. 36
prescutum/prescuta → **pretergite**/pretergites: 36, fig. 33
presternite/presternites: 37, fig. 37
presternite, **ultimate**:13, fig. 51
pre-sternum, (prehensorial) → **coxosternite**, (forcipular): 33, fig. 28
pretarsus/pretarsi: 40, fig. 43
pretarsus/pretarsi → **tarsus**/tarsi **2**: 40, fig. 44
pretarsus/pretarsi, (forcipular or prehensorial) → **tarsungulum**/tarsungula, (forcipular): 35, fig. 32
pretarsus/pretarsi, (forcipular or prehensorial) → **ungulum**/ungula, (forcipular): 35, fig. 29
pretarsus/pretarsi, (second maxillary): 32, fig. 23
pretarsus/pretarsi, **ultimate**: 43, fig. 56
pretergite/pretergites: 36, fig. 33
pretergite, **forcipular**: 32, fig. 2
pretergite, **ultimate**: 41, fig. 50
pretergum/preterga → **pretergite**/pretergites: 36, fig. 33
proarthron: 45, fig. 59
process/processes, coxal → **process**/processes, **coxopleural**: 43, fig. 52

process/processes, **coxopleural**: 43, fig. 52
process/processes, (coxosternal) **condylar**: 33
process/processes, (coxosternal or prosternal) toothed anterior → **tooth-plate**/tooth-plates, (coxosternal): 33, fig. 32
process/processes, (first maxillary) coxal → **projection**/projections, (first maxillary) **coxal**: 29, fig. 21
process/processes, (first maxillary) palpal → **lappet**/lappets, (first maxillary): 29, fig. 19
process/processes, **foraminal**: 31, fig. 20
process/processes **of** (forcipular) **trochanteroprefemur**: 35, fig. 32
process/processes of (forcipular or prehensorial) femoroid → **process**/processes **of** (forcipular) **trochanteroprefemur**: 35, fig. 32
process/processes of last coxa/coxae → **process**/processes, **coxopleural**: 43, fig. 52
process/processes (of second maxillary coxosternite), **inner**: 31, fig. 24
process/processes, **prefemoral** (spinous): 43
process/processes, (second maxillary) mesodistal → **process**/processes (of second maxillary coxosternite), **inner**: 31, fig. 24
process/processes, **spinous**: Tab. 2
process/processes, (trochanteroprefemoral) (inner spinous) → **process**/processes **of** (forcipular) **trochanteroprefemur**: 35, fig. 32
procoxa/procoxae: 37, fig. 37
production/productions (of tergite), posterior → **projection**/projections (of tergite), **posterior triangular**: 36, fig. 34
projection/projections, **anterior of the** (cephalic) **transverse suture**: 21, fig. 3
projection/projections, (first maxillary) **coxal**: 29, fig. 21
projection/projections, (first maxillary) medial → **projection**/projections, (first maxillary) **coxal**: 29, fig. 21
projection/projections, (forcipular) scapular → **point**/points, (forcipular) **scapular**: 32, fig. 27
projection/projections, posterior tergital → **projection**/projections (of tergite), **posterior triangular**: 36, fig. 34
projection/projections (of tergite), **posterior triangular**: 36, fig. 34
projection/projections **of the** (cephalic) **transverse suture**, **anterior**: 21, fig. 3
projection/projections (of tibia), **distal spinose**: 40, fig. 43
prominence/prominences, lateral prosternal → **shoulder**/shoulders (of forcipular coxosternite): 33, fig. 31
prosternite/prosternites → **presternite**/presternites: 37, fig. 37
prosternum, (prehensorial) → **coxosternite**, (forcipular): 33, fig. 28
prosubcoxa/prosubcoxae → **procoxa**/procoxae: 37, fig. 37
protarsus/protarsi → **tarsus**/tarsi **1**: 40, fig. 44
protergite/protergites → **pretergite**/pretergites: 36, fig. 33
proximotarsus/proximotarsi → **tarsus**/tarsi **1**: 40, fig. 44
pseudoporodont/pseudoporodonts → **porodont**/porodonts: 33, fig. 31
pseudosegment/pseudosegments, (tarsal) → **annulation**/annulations, (tarsal): 40, fig. 45
pulvillus/pulvilli: 27, fig. 15
punctum/puncta: Tab. 1
reticulation → **areolation**: Tab. 3
ridge/ridges, apical → **edge**/edges, **gnathal**: 26, fig. 16
ridge, (cephalic) **marginal**: 23, fig. 1
ridge/ridges (of tergite), marginal → **margination**/marginations (of tergite): 36, fig. 34
rim, (cephalic) marginal → **ridge**, (cephalic) **marginal**: 23, fig. 1
ring/rings, **circumforaminal**: 31
row/rows, ocellar → **series**/series, **ocellar**: 21
sacculus/sacculi → **pit**/pits, **carpophagus**: 39, fig. 37
saddle/saddles → **stoma-saddle**/stoma-saddles: 36, fig. 35
scape/scapes: 23, fig. 10
scapula/scapulae, (forcipular): 32, fig. 27
sclerite/sclerites, **paralabial**: 26, fig. 13
sclerite/sclerites, **postmaxillary**: 31, fig. 23
sclerotic line/lines, (prosternal) (subcondylic) → **chitin-line**/chitin-lines: 33, fig. 30
scute/scutes: 25, fig. 7
scutellum/scutella: 37, fig. 36
scutum/scuta, (dorsal) → **tergite**/tergites (of leg-bearing segment): 36, fig. 34
segment/segments 1, (gonopodal) → **article**/articles, (gonopodal) **first**: 45, fig. 61
segment/segments 2, (gonopodal) → **article**/articles, (gonopodal) **second**: 46, fig. 61
segment/segments 3, (gonopodal) → **article**/articles, (gonopodal) **third**: 46, fig. 61
segment/segments, (antennal) → **article**/articles, (antennal): 23, fig. 1

segment/segments, first tarsal → **tarsus**/tarsi **1**: 40, fig. 44
segment, **forcipular**: 32, fig. 2
segment, last leg-bearing → **segment**, **ultimate leg-bearing**: 41, fig. 49
segment, last pediferous → **segment**, **ultimate leg-bearing**: 41, fig. 49
segment/segments, (leg) → **article**/articles, (leg): 40, fig. 41
segment/segments, **leg-bearing**: 36, fig. 33
segment, maxillipede → **segment**, **forcipular**: 32, fig. 2
segment/segments, pedal → **segment**/segments, **leg-bearing**: 36, fig. 33
segment/segments, pedigerous (post-maxillipede) → **segment**/segments, **leg-bearing**: 36, fig. 33
segments, **postpedal**: 45, fig. 57
segment/segments, prehensorial → **segment**, **forcipular**: 32, fig. 2
segment/segments, second tarsal → **tarsus**/tarsi **2**: 40, fig. 44
segments, terminal → **segments**, **postpedal**: 45, fig. 57
segment, **ultimate leg-bearing**: 41, fig. 49
segment, ultimate pedal → **segment**, **ultimate leg-bearing**: 41, fig. 49
segment, ultimate pedigerous → **segment**, **ultimate leg-bearing**: 41, fig. 49
sensillum/sensilla: Tab. 2
sensillum/sensilla, trichoid → **seta**/setae: Tab. 2
series/series, **ocellar**: 21
seta/setae: Tab. 2
seta/setae, accessory → **spine**/spines, **accessory**: 41, fig. 47
seta/setae, acicular → **spine-bristle**/spine-bristles: Tab. 2
seta/setae, (leg) spiniform → **spur**/spurs, (leg): 40, fig. 44
seta/setae, **pectinal**: 40, fig. 44
seta/setae, (second maxillary) **plumose**: 32, fig. 26
seta/setae, sensory → **sensillum**/sensilla: Tab. 2
setation: Tab. 3
shaft/shafts, (mandibular) → **trunk**/trunks, (mandibular): 27, fig. 15
shank/shanks → **manubrium**/manubria: 26, fig. 16
shield, cephalic → **plate**, **cephalic**: 20, fig. 2
shield/shields, dorsal → **tergite**/tergites (of leg-bearing segment): 36, fig. 34
shield, head → **plate**, **cephalic**: 20, fig. 2
shield/shields, ventral → **sternite**/sternites (of leg-bearing segment): 37, fig. 38
shoulder/shoulders (of forcipular coxosternite): 33, fig. 31
side-piece/side-pieces, (labral): 26, fig. 13
side-piece/side-pieces, (labral) → **part**/parts, (labral) **lateral**: 26, fig. 14
sinus, (coxosternal) median → **diastema**, **median**: 33, fig. 31
sinus of mesarthron: 45, fig. 59
socket/sockets, **setal**: Tab. 1
sole-hair/sole-hairs, **resilient**: 40, fig. 45
spicula/spiculae: Tab. 2
spiculum/spicula: 23, fig. 9
spiculum/spicula → **spicula**/spiculae: Tab. 2
spina/spinae → **spine**/spines: Tab. 2
spine/spines: Tab. 2
spine/spines, **accessory**: 41, fig. 47
spine/spines, basal → **spine**/spines, **accessory**: 41, fig. 47
spine/spines, claw → **spine**/spines, **accessory**: 41, fig. 47
spine/spines, (coxopleural): 43, fig. 52
spine/spines, (coxopleural) **side**: 43
spine/spines, (coxosternal or prosternal) accessory → **porodont**/porodonts: 33, fig. 31
spine/spines, (coxosternal or prosternal) ectal → **porodont**/porodonts: 33, fig. 31
spine/spines, (coxosternal or prosternal) lateral → **porodont**/porodonts: 33, fig. 31
spine/spines, (gonopodal) basal → **spur**/spurs, (gonopodal): 46, fig. 61
spine/spines, hairlike → **spicula**/spiculae: Tab. 2
spine/spines, (leg) → **spur**/spurs, (leg): 40, fig. 44
spine/spines, parodontal → **porodont**/porodonts: 33, fig. 31
spine/spines, **prefemoral**: 43, fig. 55
spine/spines, (prefemoral) **corner**: 43

spine/spines, (prefemoral) distomedial → **spine**/spines, (prefemoral) **corner**: 43
spine/spines, (pretarsal) **posteroventral**: 41
spine/spines, (pretarsal) **subsidiary**: 41
spine/spines, sensory → **spine**/spines, **accessory**: 41, fig. 47
spine-bristle/spine-bristles: Tab. 2
spinneret → **penis**: 46, fig. 54
spinoseta/spinosetae → **spine-bristle**/spine-bristles: Tab. 2
spinula/spinulae: Tab. 2
spinulation → **plectrotaxy**: Tab. 3
spinule/spinules → **spinula**/spinulae: Tab. 2
spiracle/spiracles: 37, fig. 36
spiracle/spiracles → **stoma**/stomata: 36, fig. 35
spot/spots, clypeal → **area**/areas, clypeal: 25, fig. 7
spur/spurs: Tab. 2
spur/spurs, accessory → **spine**/spines, **accessory**: 41, fig. 47
spur/spurs, anterior accessory → **spine**/spines, **anterior accessory**: 41, fig. 47
spur/spurs, basal → **spine**/spines, **accessory**: 41, fig. 47
spur/spurs, (coxopleural) → **spine**/spines, (coxopleural): 43, fig. 52
spur/spurs, (gonopodal): 46, fig. 61
spur/spurs, (gonopodal) **supplementary**: 46
spur/spurs, (leg): 40, fig. 44
spur/spurs, posterior accessory → **spine**/spines, **posterior accessory**: 41, fig. 47
spur/spurs, prefemoral dorsal → **spine**/spines, **prefemoral**: 43, fig. 55
spur/spurs, sensory → **spine**/spines, **accessory**: 41, fig. 47
spur/spurs, tibial → **projection**/projections (of tibia), **distal spinose**: 40, fig. 43
spurulation → **plectrotaxy**: Tab. 3
statumen/statuminia: 31, fig. 24
sternite/sternites → **metasternite**/metasternites: 39, fig. 37
sternite, **first genital**: 45, fig. 54
sternite, (first maxillary): 29
sternite, genital → **sternite**, **second genital**: 45, fig. 58
sternite/sternites, intercalary → **presternite**/presternites: 37, fig. 37
sternite, **intermediate**: 45, fig. 54
sternite, last → **sternite** or **metasternite of the ultimate leg-bearing segment**: 41, fig. 51
sternite of first genital segment → **sternite**, **first genital**: 45, fig. 54
sternite/sternites (of leg-bearing segment): 37, fig. 38
sternite of second genital segment → **sternite**, **second genital**: 45, fig. 58
sternite of the last leg-bearing segment → **sternite of the ultimate leg-bearing segment**: 41, fig. 51
sternite of the last trunk segment → **sternite of the ultimate leg-bearing segment**: 41, fig. 51
sternite of the ultimate leg-bearing segment: 41, fig. 51
sternite, pregenital → **sternite**, **first genital**: 45, fig. 54
sternite, **second genital**: 45, fig. 58
sternite, ultimate (pedal) → **sternite** or **metasternite of the ultimate leg-bearing segment**: 41, fig. 51
sternobothrium/sternobothria: 39, fig. 40
sternum/sterna → **sternite**/sternites (of leg-bearing segment): 37, fig. 38
sternum, (first maxillary) → **coxosternite**, (first maxillary): 29, fig. 19
sternum, last → **sternite** or **metasternite of the ultimate leg-bearing segment**: 41, fig. 51
stigma/stigmata → **spiracle**/spiracles: 37, fig. 36
stigma/stigmata → **stoma**/stomata: 36, fig. 35
stigmatopleurite/stigmatopleurites: 37, fig. 36
stigmopleurite/stigmopleurites → **stigmatopleurite**/stigmatopleurites: 37, fig. 36
stilus/stili: 23, fig. 8
stoma/stomata: 36, fig. 35
stoma-saddle/stoma-saddles: 36, fig. 35
stomatotergite/stomatotergites: 36, fig. 35
stria/striae → **sulcus**/sulci: Tab. 1
strip, **mid-longitudinal areolate**: 25, fig. 9
stripe, mid-longitudinal areolate → **strip**, **mid-longitudinal areolate**: 25, fig. 9
style/styles, (genital): 45, fig. 60

subcoxa/subcoxae: 37
sulcus/sulci: Tab. 1
sulcus/sulci → **suture**/sutures: Tab. 1
sulcus/sulci, (cephalic) **marginal**: 23, fig. 1
sulcus, (cephalic) **median**: 20, fig. 1
sulcus/sulci, (cephalic) **paramedian**: 23, fig. 4
sulcus, frontal → **suture**, (cephalic) **transverse**: 21, figs 3,4
sulcus/sulci (of sternite), cross → **suture**/sutures (of sternite), **cruciform**: 39, fig. 39
sulcus/sulci (of sternite), cruciform → **suture**/sutures (of sternite), **cruciform**: 39, fig. 39
sulcus/sulci (of sternite), median → **sulcus**/sulci (of sternite), **median longitudinal**: 39, fig. 39
sulcus/sulci (of sternite), **median longitudinal**: 39, fig. 39
sulcus/sulci (of sternite), mid-longitudinal → **sulcus**/sulci (of sternite), **median longitudinal**: 39, fig. 39
sulcus/sulci (of sternite), **transverse**: 39, fig. 39
sulcus/sulci (of tergite), (anterior) cervical → **sulcus**/sulci **or suture**/sutures (of tergite), (anterior) **transverse**: 36, fig. 33
sulcus/sulci (of tergite), (anterior) **transverse**: 36, fig. 33
sulcus/sulci (of tergite), curved lateral → **suture**/sutures (of tergite), **oblique**: 36, fig. 33
sulcus/sulci (of tergite), **lateral crescentic**: 36
sulcus/sulci (of tergite), semi-lunar → **sulcus**/sulci **or suture**/sutures (of tergite), (anterior) **transverse**: 36, fig. 33
sulcus/sulci (of tergite), paramedian longitudinal → **sulcus**/sulci or **suture**/sutures (of tergite), **paramedian**: 36, fig. 33
sulcus/sulci (of tergite), **paramedian**: 36, fig. 33
sulcus/sulci, transverse collar → **sulcus**/sulci **or suture**/sutures (of tergite), (anterior) **transverse**: 36, fig. 33
suprascutellum/suprascutella → **paratergite**/paratergites, **intercalary**: 37, fig. 36
suprascutellum/suprascutella, primary → **paratergite**/paratergites, **primary intercalary**: 37, fig. 36
suprascutellum/suprascutella, secondary → **paratergite**/paratergites, **secondary intercalary**: 37, fig. 36
suture/sutures: Tab. 1
suture/sutures, **antennocellar**: 21, fig. 3
suture/sutures, buccal → **suture**/sutures (of cephalic pleurite), **transverse**: 23, fig. 7
suture, cephalic → **suture**, (cephalic) **transverse**: 21, figs 3,4
suture/sutures, (cephalic) **paramedian**: 23, fig. 6
suture, (cephalic) **transverse**: 21, figs 3,4
suture/sutures, clypeal → **suture**/sutures, **paraclypeal**: 25, fig. 7
suture, **clypeolabral**: 25, fig. 12
suture/sutures, (forcipular) **coxopleural**: 33, fig. 30
suture/sutures, (forcipular) **trochanteral**: 35, fig. 29
suture, frontal → **suture**, (cephalic) **transverse**: 21, figs 3,4
suture/sutures, (mandibular) **cruciform**: 27, fig. 16
suture/sutures (of cephalic pleurite), **transverse**: 23, fig. 7
suture/sutures (of sternite), **cruciform**: 219, fig. 39
suture/sutures (of sternite), **trigonal**: 39
suture/sutures (of tergite), (anterior) cervical → **suture**/sutures (of tergite), (anterior) **transverse**: 36, fig. 33
suture/sutures (of tergite), (anterior) **transverse**: 36, fig. 33
suture/sutures (of tergite), arcuate → **suture**/sutures (of tergite), **oblique**: 36, fig. 33
suture/sutures (of tergite), **cruciform**: 36, fig. 33
suture/sutures (of tergite), **lateral longitudinal**: 36, fig. 33
suture/sutures (of tergite), **oblique**: 36, fig. 33
suture/sutures (of tergite), **paramedian**: 36, fig. 33
suture/sutures (of tergite), paramedian longitudinal → **suture**/sutures (of tergite), **paramedian**: 36, fig. 33
suture/sutures (of tergite), transversal → **sulcus**/sulci **or suture**/sutures (of tergite), (anterior) **transverse**: 36, fig. 33
suture/sutures, **paraclypeal**: 25, fig. 7
suture/sutures, **parastatuminial**: 31, fig. 24
suture/sutures, pleuroprosternal → **suture**/sutures, (forcipular) **coxopleural**: 33, fig. 30
suture/sutures, (second maxillary) pleurosternal → **statumen**/statuminia: 31, fig. 24
suture/sutures, T1 ring → **sulcus**/sulci **or suture**/sutures (of tergite), (anterior) **transverse**: 36, fig. 33
suture/sutures, transbuccal → **suture**/sutures (of cephalic pleurite), **transverse**: 23, fig. 7
syncoxite, (first maxillary) → **coxosternite**, (first maxillary): 29, fig. 19
syncoxosternum, (first maxillary) → **coxosternite**, (first maxillary): 29, fig. 19
syntelopodite, (gonopodal): 45, fig. 59

tarsale/tarsalia → **annulation**/annulations, (tarsal): 40, fig. 45
tarsalium/tarsalia → **article**/articles, **tarsal**: 40
tarsomere/tarsomeres → **annulation**/annulations, (tarsal): 40, fig. 45
tarsomere/tarsomeres → **article**/articles, **tarsal**: 40
tarsomere/tarsomeres 1 → **tarsus**/tarsi **1**: 40, fig. 44
tarsomere/tarsomeres 2 → **tarsus**/tarsi **2**: 40, fig. 44
tarsungula/tarsungulae, (forcipular or prehensorial) → **tarsungulum**/tarsungula, (forcipular): 35, fig. 32
tarsungulum/tarsungula, (forcipular): 35, fig. 32
tarsus/tarsi **1**: 40, fig. 44
tarsus/tarsi **2**: 40, fig. 44
tarsus/tarsi, first → **tarsus**/tarsi **1**: 40, fig. 44
tarsus/tarsi, (forcipular): 35, fig. 29
tarsus/tarsi, (forcipular or prehensorial) → **tibia**/tibiae, (forcipular): 35, figs 29, 32
tarsus/tarsi, (leg) → **tarsus**/tarsi **1**: 40, fig. 44
tarsus/tarsi I → **tarsus**/tarsi **1**: 40, fig. 44
tarsus/tarsi, I → **tarsus**/tarsi **1**: 40, fig. 44
tarsus/tarsi II → **tarsus**/tarsi **2**: 40, fig. 44
tarsus/tarsi, II → **tarsus**/tarsi **2**: 40, fig. 44
tarsus/tarsi, (leg): 40, fig. 43
tarsus/tarsi, second → **tarsus**/tarsi **2**: 40, fig. 44
tarsus/tarsi, (second maxillary): 31, fig. 25
tarsus/tarsi, (second maxillary) → **article**/articles **3**, (second maxillary): 32, fig. 23
telomere/telomeres 1, (second maxillary) → **article**/articles **1**, (second maxillary): 32, fig. 23
telomere/telomeres 2, (second maxillary) → **article**/articles **2**, (second maxillary): 32, fig. 23
telomere/telomeres 3, (second maxillary) → **article**/articles **3**, (second maxillary): 32, fig. 23
telopod/telopods, (second maxillary) → **telopodite**/telopodites, (second maxillary): 31, fig. 21
telopodite/telopodites, (first maxillary): 29, fig. 21
telopodite/telopodites, forcipular → **forcipule**/forcipules: 35, fig. 28
telopodite/telopodites, (gonopodal): 45, fig. 61
telopodite/telopodites, prehensorial → **forcipule**/forcipules: 35, fig. 28
telopodite/telopodites, (second maxillary): 31, fig. 21
telotarsus/telotarsi → **tarsus**/tarsi **2**: 40, fig. 44
tenaciped/tenacipeds: 39
tentorium/tentoria: 26, fig. 13
tergite/tergites → **metatergite**/metatergites: 36, fig. 33
tergite, anal → **tergite**, **postpedal**: 46, fig. 49
tergite, **first genital**: 45, fig. 57
tergite, **forcipular**: 32, fig. 2
tergite/tergites, intercalary → **pretergite**/pretergites: 36, fig. 33
tergite, **intermediate**: 45, fig. 57
tergite, last → **tergite** or **metatergite of the ultimate leg-bearing segment**: 41, fig. 49
tergite/tergites (of leg-bearing segment): 36, fig. 34
tergite of telson → **tergite**, **postpedal**: 46, fig. 49
tergite of the ultimate leg-bearing segment: 41, fig. 49
tergite, **postpedal**: 46, fig. 49
tergite, ultimate → **tergite** or **metatergite of the ultimate leg-bearing segment**: 41, fig. 49
tergum/terga → **tergite**/tergites (of leg-bearing segment): 36, fig. 34
tergum of postpedal segments → **tergite**, **postpedal**: 46, fig. 49
termination/terminations of marginal ridge, lateral → **interruption**/interruptions (of cephalic plate), **lateral marginal**: 23
tibia/tibiae, (forcipular): 35, figs 29, 32
tibia/tibiae, (forcipular or prehensorial) → **femur**/femora, (forcipular): 35, figs 29, 32
tibia/tibiae, (leg): 40, fig. 43
tibia/tibiae, (second maxillary): 31, fig. 25
tibia/tibiae, (second maxillary) → **article**/articles **2**, (second maxillary): 32, fig. 23
tibioid/tibioids, (forcipular or prehensorial) → **tibia**/tibiae, (forcipular): 35, figs 29, 32
tibio-tarsus/tibio-tarsi, (first maxillary) → **article**/articles, (first maxillary), **distal**: 29, fig. 18
tooth/teeth, (coxosternal): 33, fig. 32
tooth/teeth, (coxosternal) → **denticle**/denticles, (coxosternal): 33, fig. 30
tooth/teeth, (forcipular or prehensorial) → **denticle**/denticles, (forcipular): 35, figs 27, 30

tooth/teeth, (labral) → **denticle**/denticles, (labral): 26, fig. 12
tooth/teeth, (labral) → **tubercle**/tubercles, (labral): 26, fig. 14
tooth, (labral) **mid-piece**: 26, fig. 13
tooth/teeth, (mandibular): 27, fig. 15
tooth/teeth, (mandibular) **basal**: 27
tooth/teeth, (mandibular) **tricuspid**: 27, fig. 16
tooth/teeth (of tarsungulum), basal → **denticle**/denticles (of tarsungulum), **basal**: 35, fig. 30
tooth/teeth, prefemoral → **spine**/spines, **prefemoral**: 43, fig. 55
tooth/teeth, (prosternal or forcipular) → **tooth**/teeth, (forcipular coxosternal): 33, fig. 32
tooth/teeth**, saw**: 43, fig. 56
tooth/teeth, saw-like → **tooth**/teeth, **saw**: 43, fig. 56
tooth/teeth, (trochanteroprefemoral) (inner or median) → **process**/processes **of** (forcipular) **trochanteroprefemur**: 35, fig. 32
tooth-plate/tooth-plates, (coxosternal): 33, fig. 32
toxicognath/toxicognaths → **forcipule**/forcipules: 35, fig. 28
trochanter/trochanters, (leg): 40, fig. 38
trochanter/trochanters, (second maxillary): 31, fig. 25
trochanteroprefemur/trochanteroprefemora, (forcipular): 35, figs 29, 32
trunk/trunks, (mandibular): 27, fig. 15
trunk-segment/trunk-segments → **segment**/segments, **leg-bearing**: 36, fig. 33
trunk-segment, last → **segment**, **ultimate leg-bearing**: 41, fig. 49
tubercle/tubercles: Tab. 2
tubercle/tubercles, distomedial prefemoral → **process**/processes, **prefemoral** (spinous): 43
tubercle/tubercles, (labral): 26, fig. 12
tubercle/tubercles, (prosternal or forcipular) (marginal) → **tooth**/teeth, (forcipular coxosternal): 33, fig. 32
unguis/ungues → **unguis**/ungues **proper**: 41, fig. 47
unguis/ungues **proper**: 41, fig. 47
ungula/ungulae, (forcipular or prehensorial) → **ungulum**/ungula, (forcipular): 35, fig. 29
ungulum/ungula, (forcipular): 35, fig. 29
valve/valves, **anal**: 46, fig. 58
vestiture → **setation**: Tab. 3
